# Supplementary material for: A stable zirconium based metal-organic framework for specific recognition of representative polychlorinated dibenzo-p-dioxin molecules
Source: Nat Commun. 2019 Aug 27;10:3861. doi: 10.1038/s41467-019-11912-4 (PMC6712023; doi:10.1038/s41467-019-11912-4)
Supplement: Supplementary file 1 — Supplementary Information [file 41467_2019_11912_MOESM1_ESM.pdf]

Supplementary Information

A stable zirconium based metal-organic framework for specific recognition of representative polychlorinated dibenzo-*p*-dioxin molecules

Wang et al.

Supplementary Methods

Material characterization. TGA data were obtained on a TGA-50 (SHIMADZU) thermogravimetric analyzer with a heating rate of 5 °C min^{-1} under air atmosphere. N_2 adsorption/desorption isotherms were measured by the volumetric method using a Micromeritics ASAP2020 surface area and pore analyzer at 77 K. The PXRD patterns were recorded on a Rigaku Smartlab3 X-ray Powder Diffractometer equipped with a Cu sealed tube (λ = 1.54178 Å) at room temperature. Simulation of the PXRD pattern was carried out by the single-crystal data and diffraction-crystal module of the Mercury program available free of charge via internet at http://www.ccdc.cam.ac.uk/mercury/. The photoluminescence (PL) spectra were recorded in a Hitachi F-7000 fluorescence spectrophotometer at room temperature. UV-Vis spectra were obtained with a UV-2600 spectrophotometer in the range of 250-800 nm at room temperature. The luminescence lifetimes were measured in an FLS980 fluorescence spectrophotometer. The concentrations of BCDD and TCDD in aqueous solutions were recorded using Shimadzu GCMS-TQ8050 gas chromatograph mass spectrometer.

Preparation of UiO-66: $ZrCl_4$ (930 mg, 4 mmol) and 1,4-benzenedicarboxylic acid (H_2BDC, 1.32 g, 8 mmol) were dissolved in a mixed solution of DMF (24 mL) and concentrated HCl (0.67 mL). Then the solution was heated inside a 100 ml autoclave at 220 ^{o}C for 16 h. After the reaction system cooling down, the white powder product was harvested through centrifuge. The materials were washed with DMF and methanol with a Soxhlet extractor for 2 and 3 days, respectively (yield: 850 mg).

Preparation of BUT-39: $ZrCl_4$ (0.03 mmol, 7.5 mg) and benzoic acid (320 mg) were

dissolved in DMF (1 mL) containing trifluoroacetic acid (10 μL). The solution stood at room temperature for 1 h, and H_3BTBA (0.01 mmol, 5 mg) was ultrasonically dissolved in DMF (1 mL). Then, the two solutions were mixed in a 4 mL vial. The mixture was sealed and heated in a 120 °C oven for 5 days. After cooling to room temperature, the colorless hexagonal crystals were collected by filtration and washed with DMF and acetone, and then dried in air (yield: 6.0 mg).

Preparation of BUT-12: $ZrCl_4$ (48 mg, 0.2 mmol), H_3CTTA (40 mg, 0.08 mmol), and formic acid (8 mL) were ultrasonically dissolved in *N,N'*-dimethylformamide (DMF, 8 mL) in a 20 mL Pyrex vial. The vial was sealed and then heated at 120 °C for 48 h in an oven. After cooling to room temperature, the resulting colorless crystals were harvested by filtration and washed with DMF and acetone, and then dried in air (yield: 42 mg).

Preparation of BUT-66: In a 5 mL glass vial, $ZrOCl_2 \cdot 8H_2O$ (0.0322 g, 0.1 mmol) and H_2BDB (0.0636 g, 0.2 mmol) were added in a mixture of 1.5 mL of DMF and 0.25 mL of acetic acid. The vial was then sealed and kept under ultrasonication for about 30 min to dissolve all the starting materials. The mixture was heated at 120 °C for 24 h and then cooled down to room temperature. Colorless hexagonal crystals were obtained by filtration. After the as-synthesized crystals were guest exchanged with methanol for 3 days (20 mL 3 3) and subsequently evacuated at 80 °C under high vacuum, 0.035 g (yield ca. 81% based on Zr) of the final product of BUT-66 was obtained.

Preparation of BUT-15: $ZrCl_4$ (48 mg, 0.20 mmol), $H_4PBPTTBA$ (42 mg, 0.06 mmol), and acetic acid (8 mL) were ultrasonically dissolved in DMF (10 mL) in a 20 mL Pyrex vial and sealed. The vial was then heated at 120 °C for 48 h in an oven. After cooling to room

temperature, the resulting yellow crystals were harvested by filtration and washed with DMF and acetone, and then dried in air (yield: 42 mg).

Preparation of NU-1000: $ZrCl_4$ (70 mg, 0.3 mmol), H_4TBAPy (40 mg, 0.06 mmol), and benzoic acid (2.7 g) were ultrasonically dissolved in DMF (8 mL) in a 20 mL Pyrex vial and sealed. The vial was then heated at 120 ^oC for 48 h in an oven. After cooling to room temperature, the resulting yellow crystals were harvested by filtration and washed with DMF and acetone, and then dried in air (yield: 40 mg).

Preparation of NU-1003: $ZrOCl_2$ (20 mg, 0.062 mmol), H_4PTTNA (8 mg, 0.09 mmol), and benzoic acid (250 mg) were ultrasonically dissolved in DMF (8 mL) in a 20 mL Pyrex vial and sealed. The vial was then heated at 120 ^oC for 24 h in an oven. After cooling to room temperature, the resulting light-yellow powder were harvested by filtration and washed with DMF and acetone, and then dried in air (yield: 11 mg).

Preparation of H_4CPTTA ligand:

A. Dimethyl 5-(4,4,5,5-tetramethyl-1,3,2-dioxaborolan-2-yl)isophthalate (1). To a 500 mL three-necked, round-bottomed flask, dimethyl 5-bromoisophthalate (10.0 g, 36 mmol), 4,4,4',4',5,5,5',5'-octamethyl-2,2'-bi(1,3,2-dioxaborolane) (18.6 g, 72 mmol), KOAc (7.8 g, 80.0 mmol), and $PdCl_2$(dppf) (400 mg) was added. The flask was connected to Schlenk line and evacuated air and refilled with the nitrogen. 350 mL of 1,4-dioxane was degassed (two hours) and added through a canula. The flask was equipped with a water condenser and reflux under the nitrogen for 24 h. After cooling to room temperature, 150 mL of H_2O was added and then extract with CH_2Cl_2. The organic phase was dried with $MgSO_4$. After removed the CH_2Cl_2 solvent, the crude product was column chromatographed over silica gel using the

petroleum : ethyl acetate = 20 : 1 as eluent to give the product as white solid in ~93% yield (10.2 g) based on dimethyl 5-bromoisophthalate. ^{1}H NMR (400 MHz, DMSO-d^{6}): δ 8.55 (s, 1H), 8.43 (s, 2H), 3.91 (s, 6H), 1.34 (s, 12H).

B. Dimethyl 3',5'-dibromo-[1,1'-biphenyl]-3,5-dicarboxylate (2). To a 500 mL three-necked, round-bottomed flask, 1,3-dibromo-5-iodobenzene (10.0 g, 27 mmol), **1** (17.7 g, 55.0 mmol), K_3PO_4 (15.0 g, 70.0 mmol), and $Pd(PPh_3)_4$ (400 mg) was added. The flask was connected to Schlenk line and evacuated air and refilled with the nitrogen. 300 mL of 1,4-dioxane and 10 mL H_2O was degassed (two hours) and added through a canula. The flask was equipped with a water condenser and react at 60 ^{o}C under the nitrogen for 24 h. The solvent was evaporated on rotary evaporator. 150 mL of H_2O was added and then extract with CH_2Cl_2. The organic phase was dried with $MgSO_4$. After removed the CH_2Cl_2 solvent, the crude product was column chromatographed over silica gel using petroleum : ethyl acetate = 20 : 1 as eluent to give the product as white solid in ~82% yield (9.6 g) based on 1,3-dibromo-5-iodobenzene. ^{1}H NMR (400 MHz, $CDCl_3$): δ 8.69 (s, 1H), 8.62 (s, 1H), 8.57 (d, 2H), 8.31 (d, 2H), 3.92 (s, 6H).

C. Trimethyl 5'-(4-(methoxycarbonyl)phenyl)-[1,1':3',1''-terphenyl]-3,4'',5-tricarboxylate (3). To a 500 mL three-necked, round-bottomed flask, **2** (5.0 g, 11 mmol), methyl 4-(4,4,5,5-tetramethyl-1,3,2-dioxaborolan-2-yl)benzoate (9.2 g, 35.0 mmol), K_3PO_4 (15.0 g, 70.0 mmol), and $Pd(PPh_3)_4$ (300 mg) was added. The flask was connected to Schlenk line and evacuated air and refilled with the nitrogen. 300 mL of 1,4-dioxane and 10 mL H_2O was degassed (two hours) and added through a canula. The flask was equipped with a water condenser and reflux under the nitrogen for 24 h. The solvent was evaporated on rotary

evaporator. 150 mL of H_2O was added and then extract with CH_2Cl_2. The organic phase was dried with $MgSO_4$. After removal of the CH_2Cl_2 solvent, the crude product was column chromatographed over silica gel using petroleum : ethyl acetate = 10 : 1 to give the product as white solid in ~73% yield (4.8 g) based on **2**. ^{1}H NMR (400 MHz, $CDCl_3$): δ 8.75 (s, 1H), 8.58 (s, 2H), 8.20 (d, 4H), 7.91 (s, 3H), 7.81 (d, 4H), 4.01 (d, 12H).

D. 5'-(4-carboxyphenyl)-[1,1':3',1''-terphenyl]-3,4'',5-tricarboxylic acid (H_4CPTTA). 3 (4.0 g) was suspended in a mixture of THF (120 mL) and MeOH (120 mL), to which 50 mL of 10 M NaOH aqueous solution was added. The mixture was stirred under reflux overnight and the THF and MeOH were removed under a vacuum. Dilute HCl was added to the remaining aqueous solution until the solution was at pH = 2. The solid was collected by filtration, washed with water, and dried to give the product as white solid (3.2 g, 90% yield).

Single-crystal X-ray diffraction. The crystal data of BUT-17 and BUT-17@BCDD were collected on a Rigaku Supernova CCD diffractometer equipped with a graphite-monochromatic enhanced Cu Kα radiation (λ = 1.54184 Å) at 100 K. The datasets were corrected by empirical absorption correction using spherical harmonics, implemented in the *SCALE3 ABSPACk* scaling algorithm. The structure of the two MOFs were solved by direct methods and refined by full-matrix least-squares on F^2 with anisotropic displacement using the *SHELXTL* software package. Non-hydrogen atoms were refined with anisotropic displacement parameters during the final cycles. Hydrogen atoms of the ligands were calculated in ideal positions with isotropic displacement parameters. Those in OH groups and coordinated H_2O molecules of the Zr-based clusters were not added but were calculated into molecular formula of the crystal data. There is large solvent accessible pore volume in the

crystals of BUT-17, which are occupied by highly disordered solvent molecules. No satisfactory disorder model for these solvent molecules could be assigned, and therefore the *SQUEEZE* program implemented in *PLATON* was used to remove the electron densities of these disordered species. Thus, all of electron densities from free solvent molecules have been "squeezed" out. The details of structural refinement can be found in Supplementary Table 1 and 2.

Activation of the as-synthesized Zr-MOFs. As-synthesized Zr-MOF samples were firstly soaked in fresh DMF for 24 h and then the extract was discarded. Fresh acetone was subsequently added, and the sample was allowed to stay in it for 8 h. This procedure was repeated three times over one day. After decanting the acetone extract, the sample was dried under a dynamic vacuum (< 10^{-3} Torr) at room temperature for 3 h. Before adsorption and fluorescence measurements, the sample was further activated using the "outgas" function of the adsorption analyzer for 10 h at 100 ^{o}C.

Adsorption experiments. Acetone aqueous solution (water : acetone = 1 : 1) of BCDD or TCDD with the concentration of 10 ppm was accurately prepared and 0.5 mL of such solution was added in a vial (1.5 mL). The solvent in the vial was dried using N_2 flow, and 0.5 mL of hexane was further added. The vial was placed on the autosampler tray and 1 μL of the extract was automatically retrieved and injected into GC-MS system for analysis. The area of the peak represents BCDD or TCDD in GC-MS was initially recorded. Then, 15 mg powder of BUT-17 was added in the above-mentioned BCDD or TCDD acetone aqueous solution (10 mL). After adsorption for 2 h at 298 K, the MOF powder was centrifuged, and the filtrate (0.5 mL) was added in a vial (1.5 mL). Following the same procedure as mentioned above, the

area of the peak represents the BCDD or TCDD in GC-MS was recorded again. The removing efficiency was thus calculated based on the changes of the areas before and after adsorption.

Cyclic tests. The used sample was centrifuged and washed several times with acetone through soaking under stirring at room temperature. After centrifugation, the wet sample was dried in a 100 ^{o}C oven for 2 hours to remove the residual solvents. The regenerated sample was used again for the detection of BCDD and TCDD.

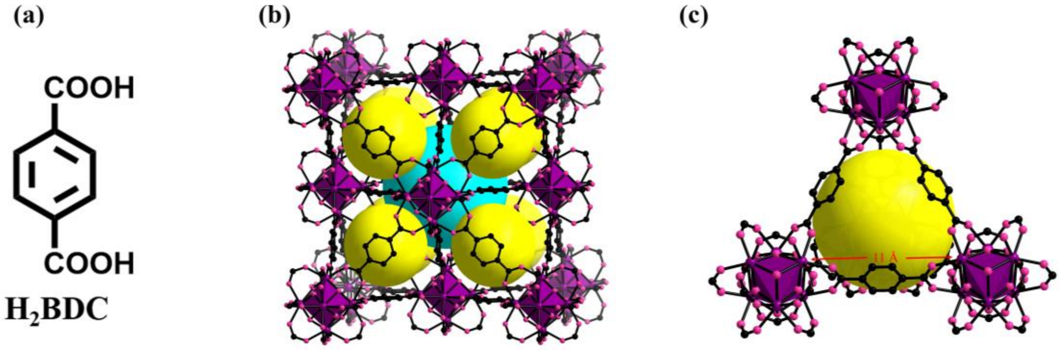

Supplementary Figure 1. Construction of UiO-66. (a) H_2BDC ligand, (b) framework structure, and (c) tetrahedral cage in UiO-66 (Color code: C, black; O, magenta; and Zr, violet; H atoms on ligands are omitted for clarity; the large yellow and blue spheres represent cage void regions inside the frameworks).

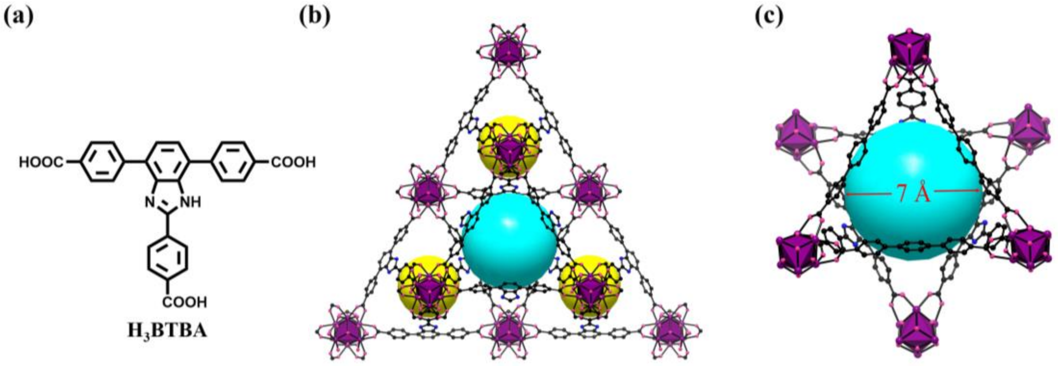

Supplementary Figure 2. Construction of BUT-39. (a) H_3BTBA, (b) framework structure, and (c) tetrahedral cage in BUT-39 (Color code: C, black; O, magenta; N, blue; and Zr, violet; H atoms on ligands are omitted for clarity; the large yellow, and blue spheres represent cage void regions inside the frameworks).

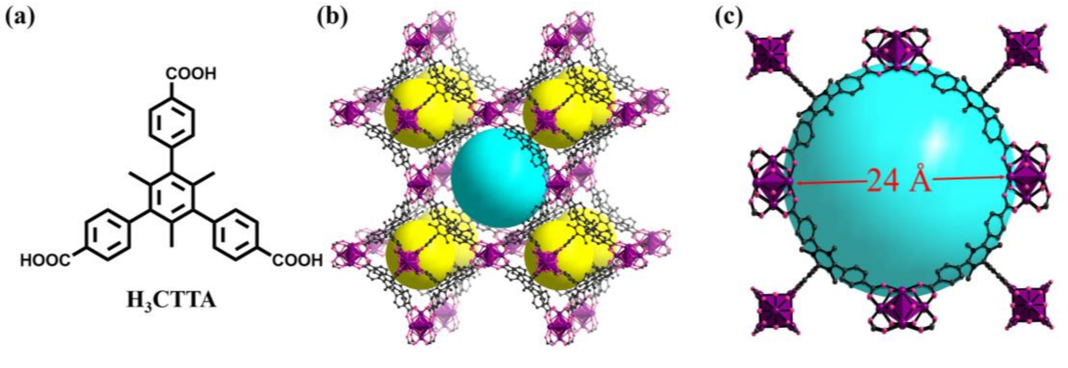

Supplementary Figure 3. Construction of BUT-12. (a) H_3CTTA ligand, (b) framework structure, and (c) cuboctahedral cage in BUT-12 (Color code: C, black; O, magenta; and Zr,

violet; H atoms on ligands are omitted for clarity; the large yellow, and green spheres represent cage void regions inside the frameworks).

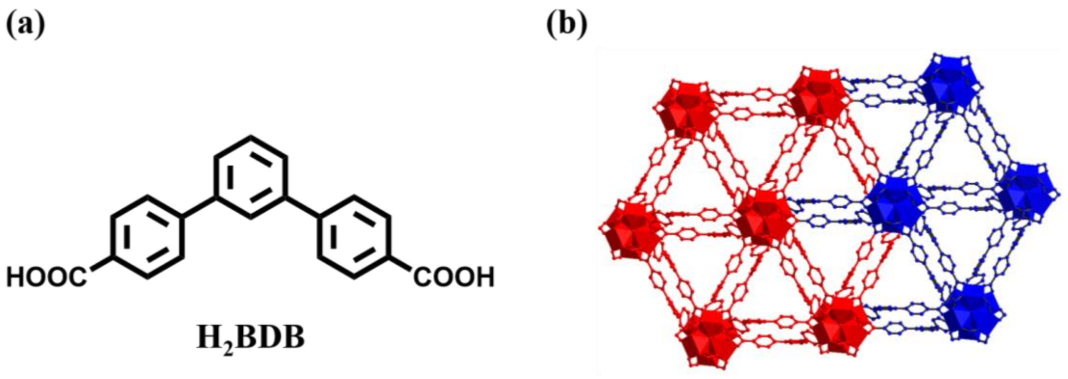

Supplementary Figure 4. Construction of BUT-66. (a) H_2BDB, (b) framework structure of BUT-66 along *c* axis.

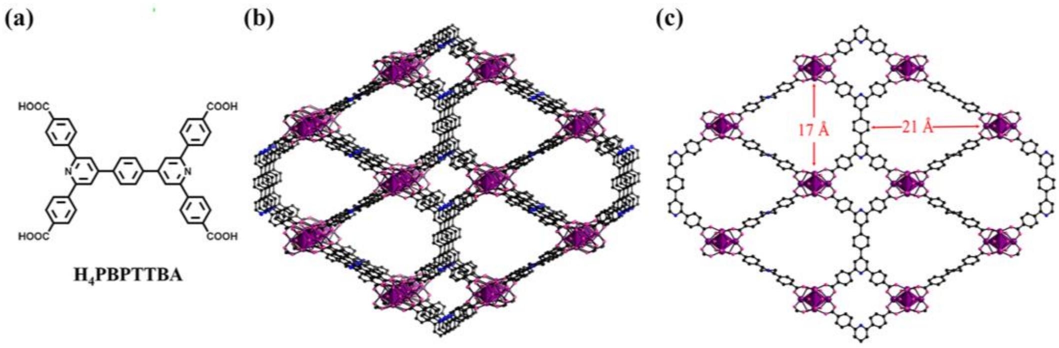

Supplementary Figure 5. Construction of BUT-15. (a) $H_4PBPTTBA$ ligand, (b) framework structure, and (c) 1D channel along *c* axis (Color code: C, black; O, magenta; and Zr, violet; H atoms on ligands are omitted for clarity).

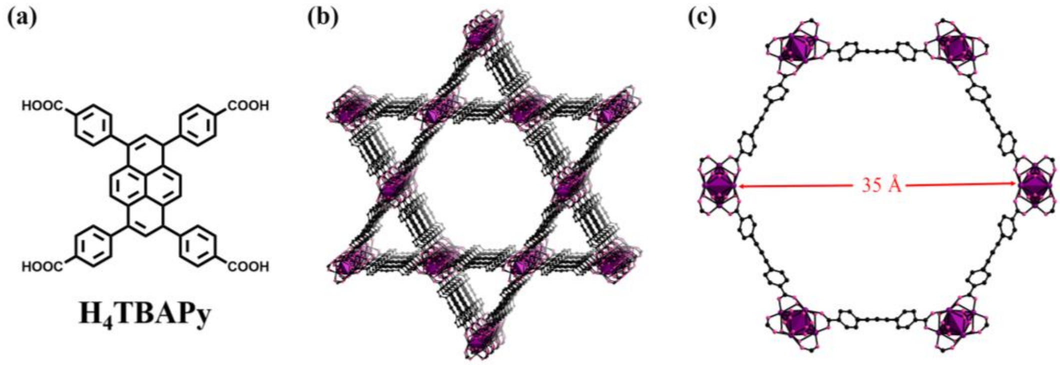

Supplementary Figure 6. Construction of NU-1000. (a) H_4TBAPy ligand, (b) framework structure, and (c) hexagonal channel along *c* axis (Color code: C, black; O, magenta; and Zr, violet; H atoms on ligands are omitted for clarity).

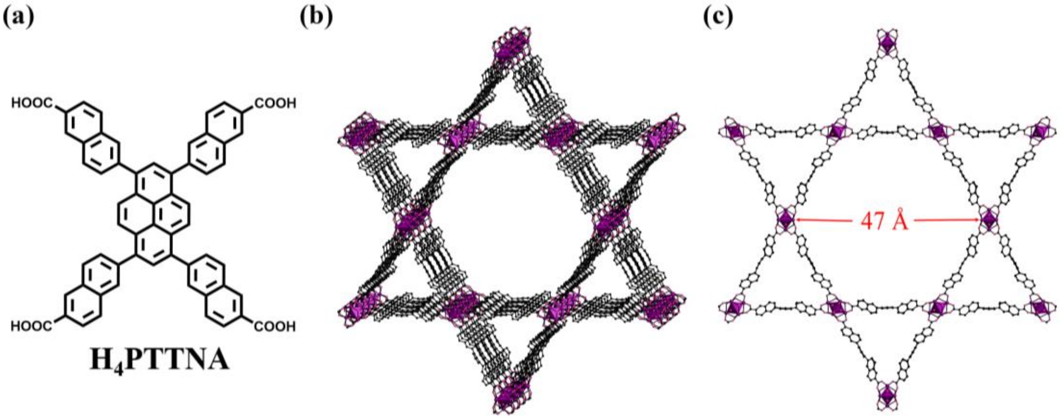

Supplementary Figure 7. Construction of NU-1003. (a) H_4PTTNA ligand, (b) framework structure, and (c) hexagonal channel along *c* axis (Color code: C, black; O, magenta; and Zr, violet; H atoms on ligands are omitted for clarity).

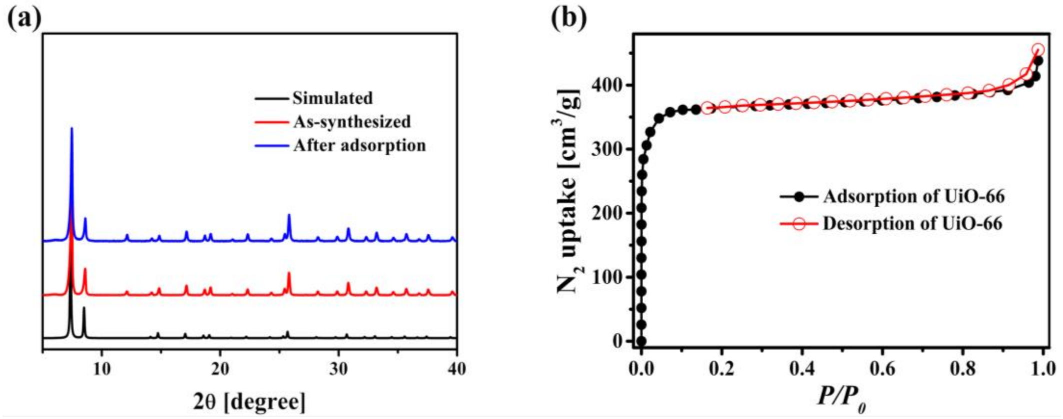

Supplementary Figure 8. Characterizations of UiO-66. (a) PXRD patterns and (b) N_2 adsorption/desorption isotherms at 77 K.

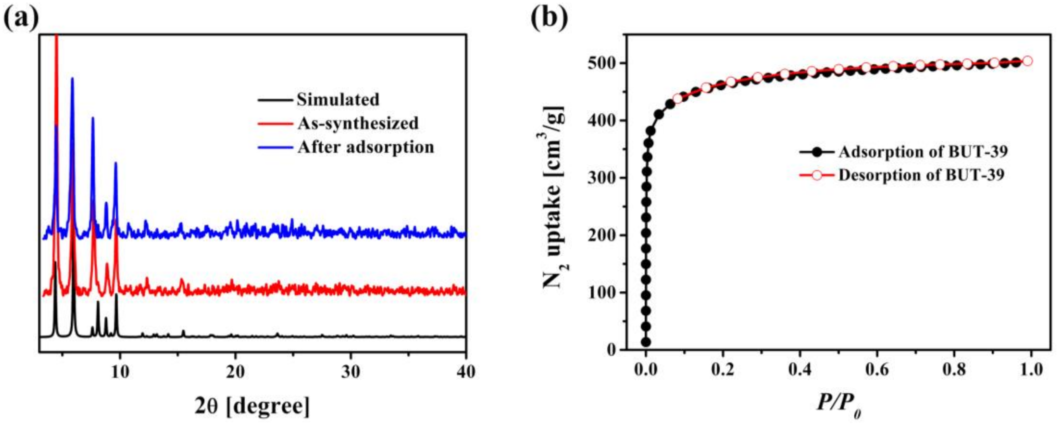

Supplementary Figure 9. Characterizations of BUT-39. (a) PXRD patterns and (b) N_2 adsorption/desorption isotherms at 77 K.

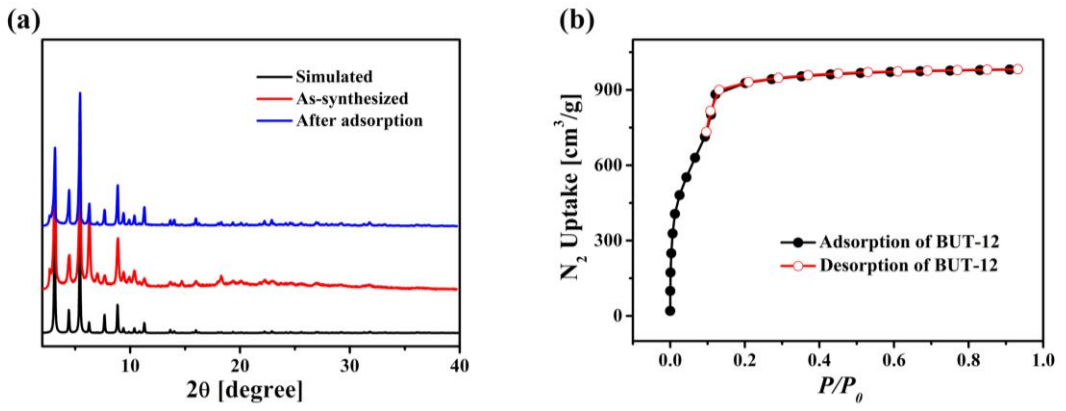

Supplementary Figure 10. Characterizations of BUT-12. (a) PXRD patterns and (b) N_2 adsorption/desorption isotherms at 77 K.

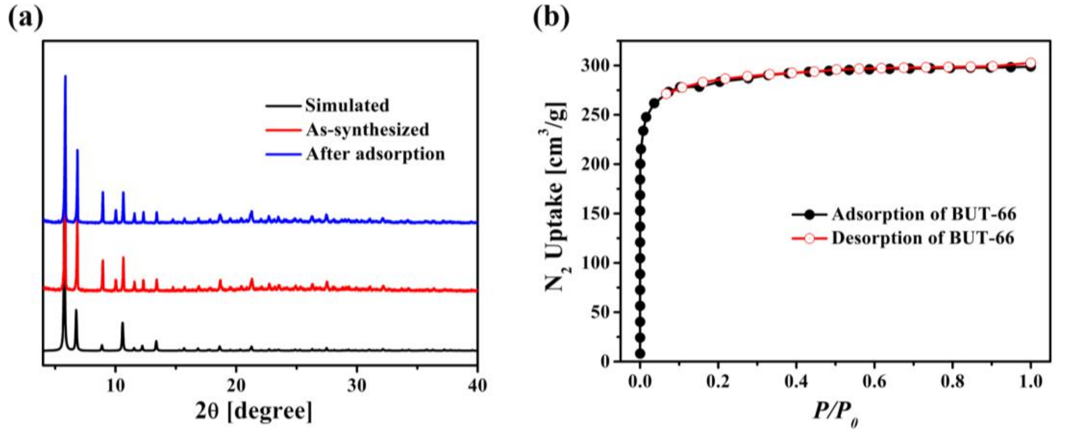

Supplementary Figure 11. Characterizations of BUT-66. (a) PXRD patterns and (b) N_2 adsorption/desorption isotherms at 77 K.

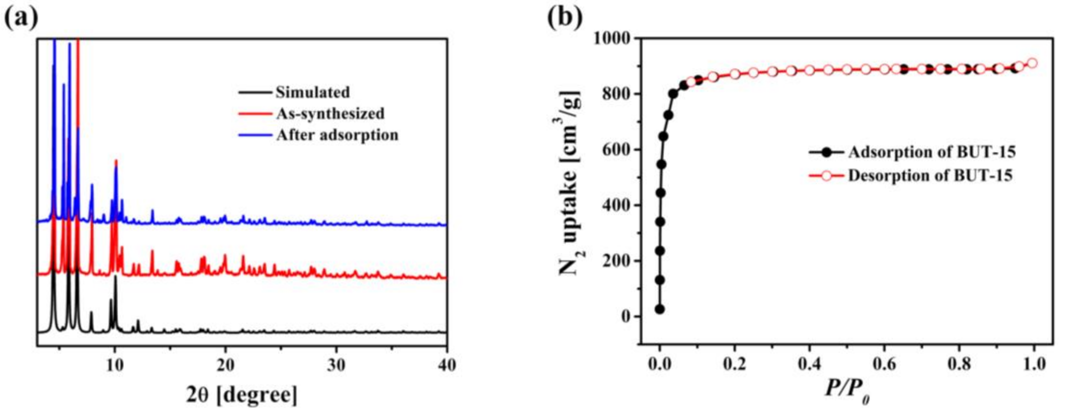

Supplementary Figure 12. Characterizations of BUT-15. (a) PXRD patterns and (b) N_2 adsorption/desorption isotherms at 77 K.

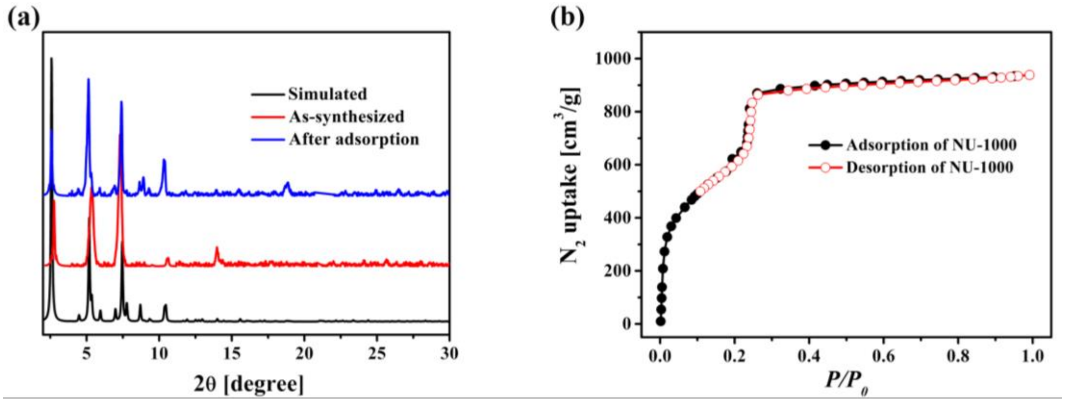

Supplementary Figure 13. Characterizations of NU-1000. (a) PXRD patterns and (b) N_2 adsorption/desorption isotherms at 77 K.

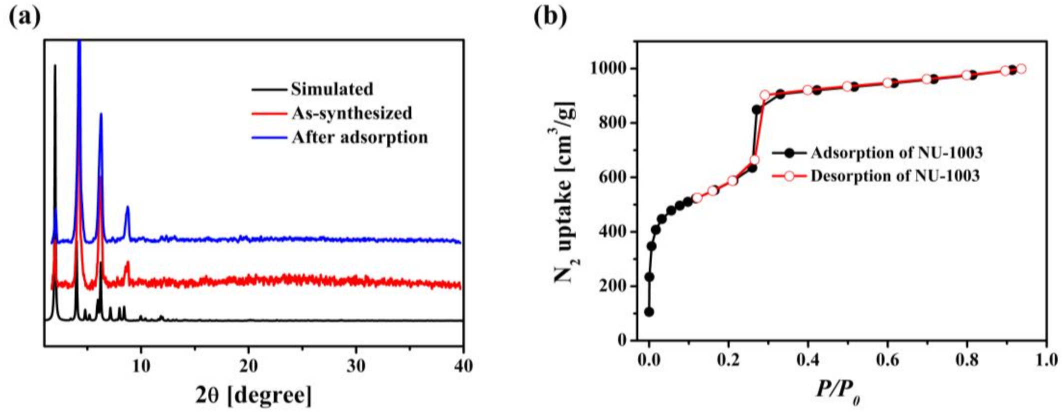

Supplementary Figure 14. Characterizations of NU-1003. (a) PXRD patterns and (b) N_2 adsorption/desorption isotherms at 77 K.

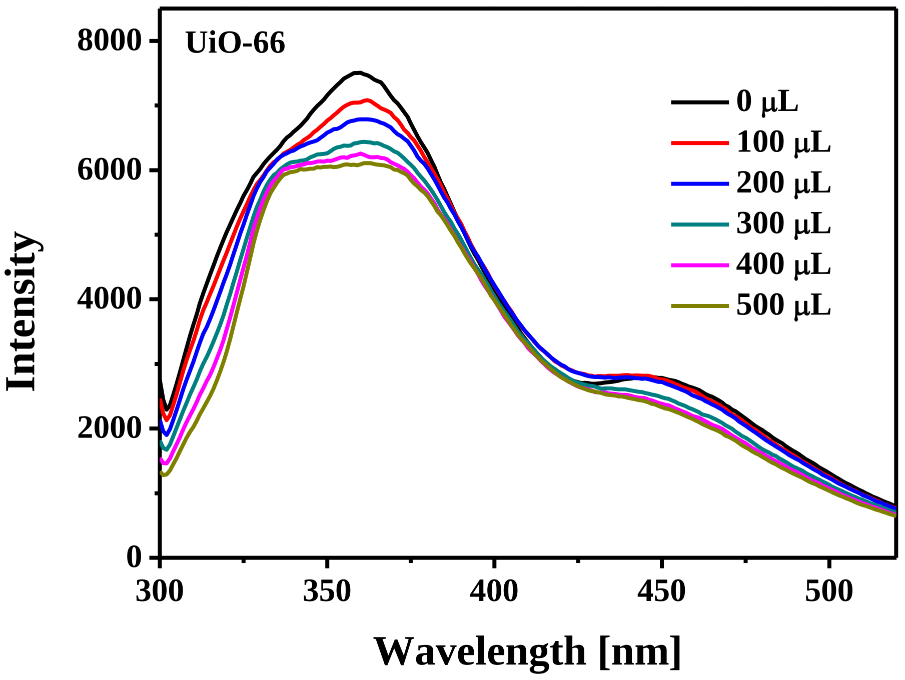

Supplementary Figure 15. Fluorescence-quenching titrations of UiO-66 by BCDD. Effect on the emission spectra of UiO-66 dispersed in hexane upon incremental addition hexane solutions of BCDD (100 ppm).

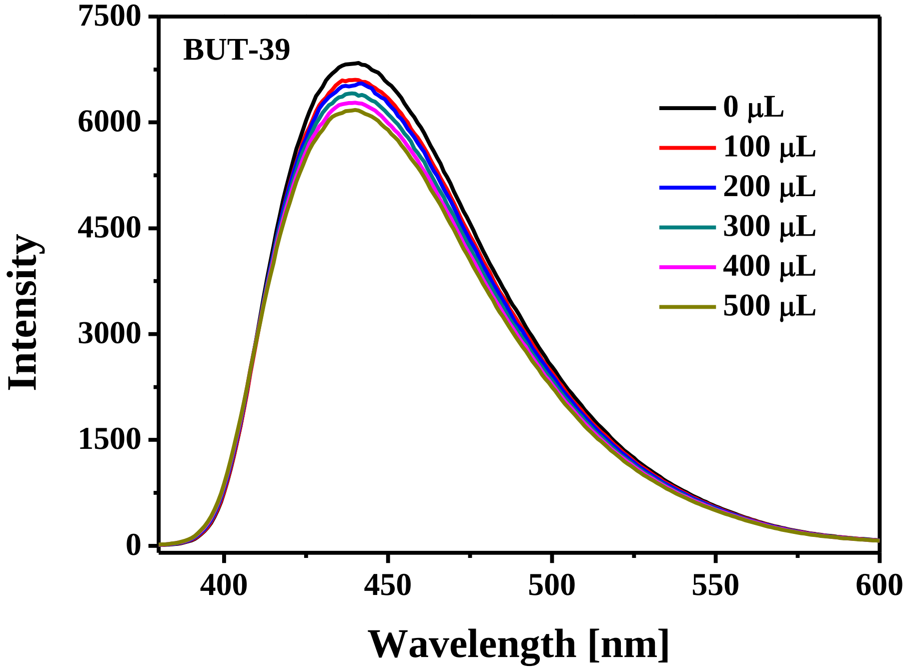

Supplementary Figure 16. Fluorescence-quenching titrations of BUT-39 by BCDD. Effect on the emission spectra of BUT-39 dispersed in hexane upon incremental addition hexane solutions of BCDD (100 ppm).

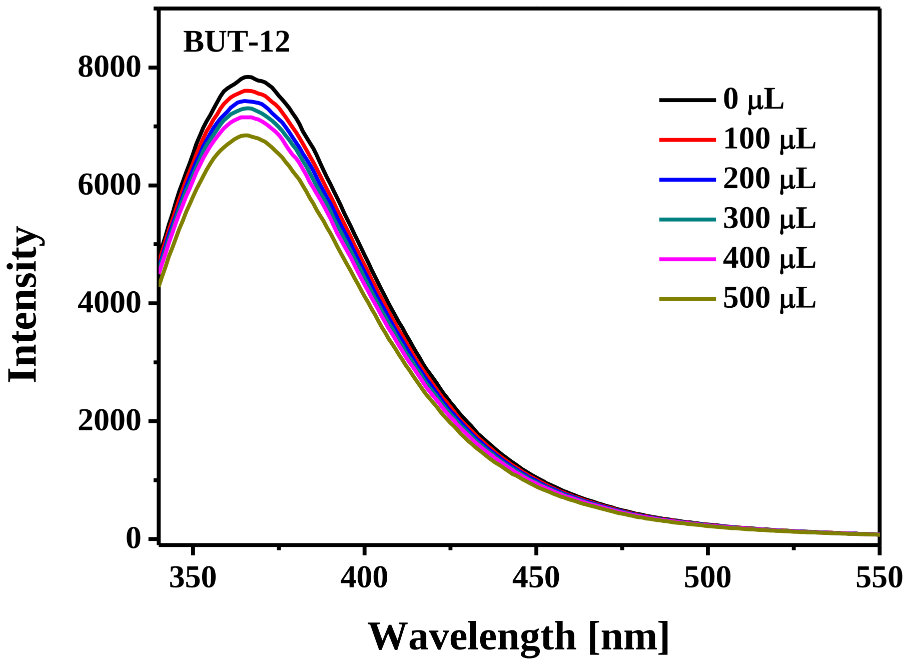

Supplementary Figure 17. Fluorescence-quenching titrations of BUT-12 by BCDD. Effect on the emission spectra of BUT-12 dispersed in hexane upon incremental addition hexane solutions of BCDD (100 ppm).

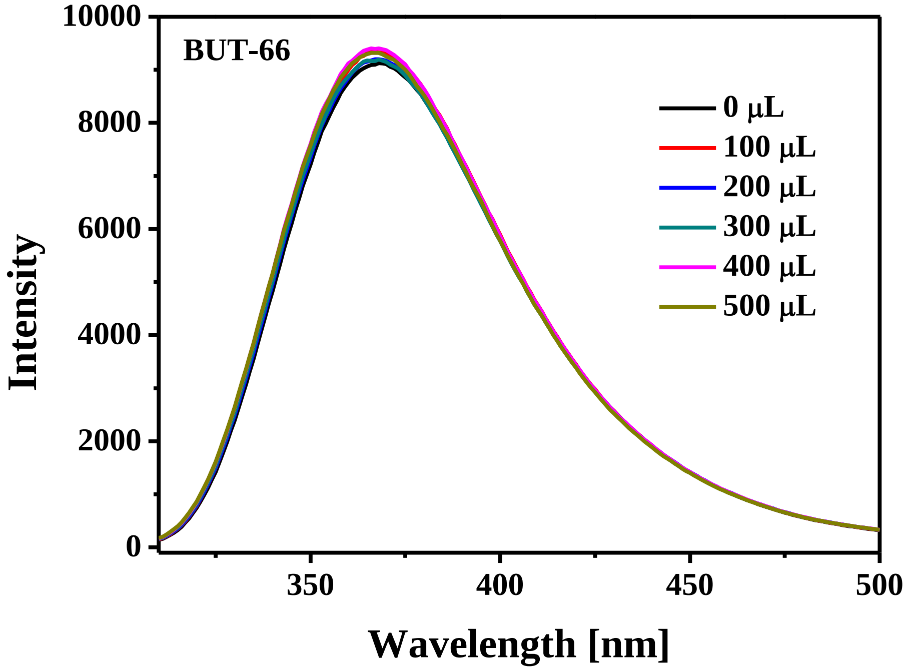

Supplementary Figure 18. Fluorescence-quenching titrations of BUT-66 by BCDD. Effect on the emission spectra of BUT-66 dispersed in hexane upon incremental addition hexane solutions of BCDD (100 ppm).

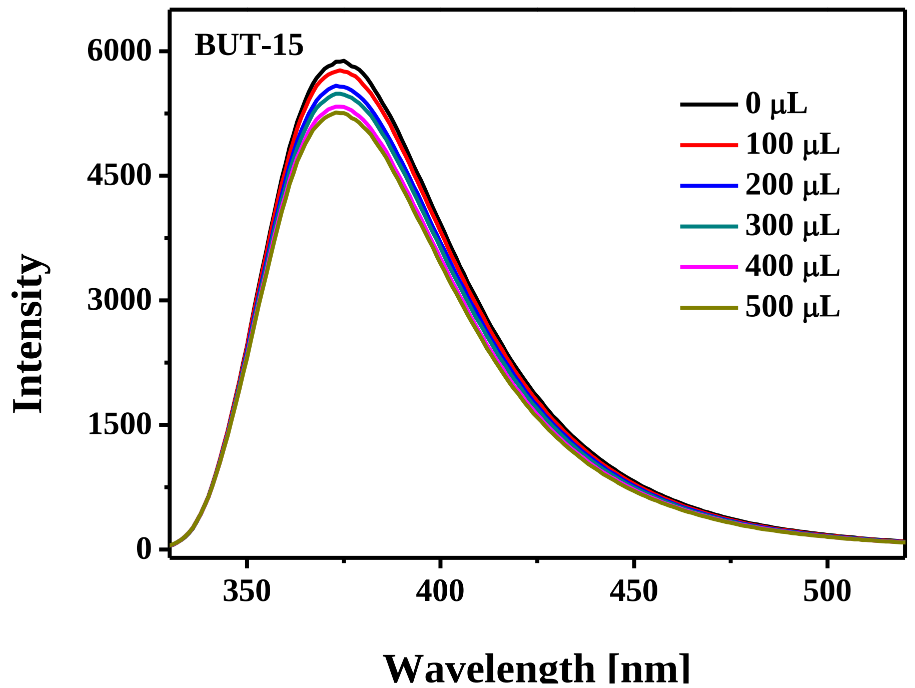

Supplementary Figure 19. Fluorescence-quenching titrations of BUT-15 by BCDD. Effect on the emission spectra of BUT-15 dispersed in hexane upon incremental addition hexane solutions of BCDD (100 ppm).

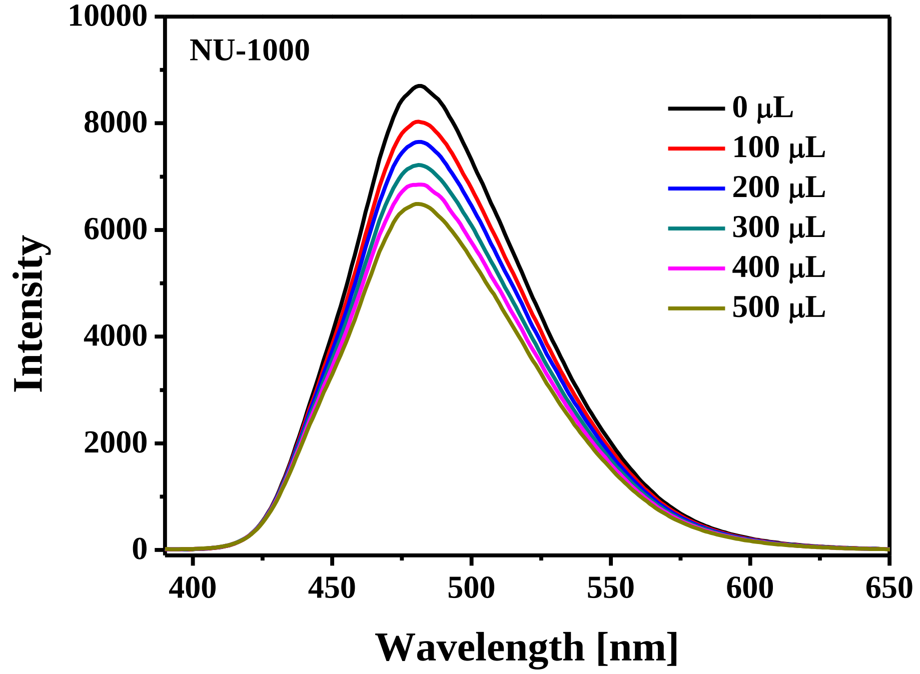

Supplementary Figure 20. Fluorescence-quenching titrations of NU-1000 by BCDD. Effect on the emission spectra of NU-1000 dispersed in hexane upon incremental addition hexane solutions of BCDD (100 ppm).

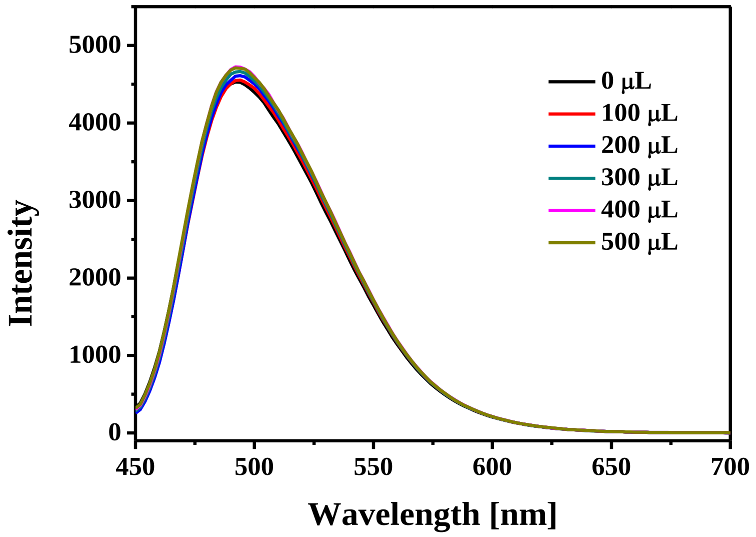

Supplementary Figure 21. Fluorescence-quenching titrations of NU-1003 by BCDD. Effect on the emission spectra of NU-1003 dispersed in hexane upon incremental addition hexane solutions of BCDD (100 ppm).

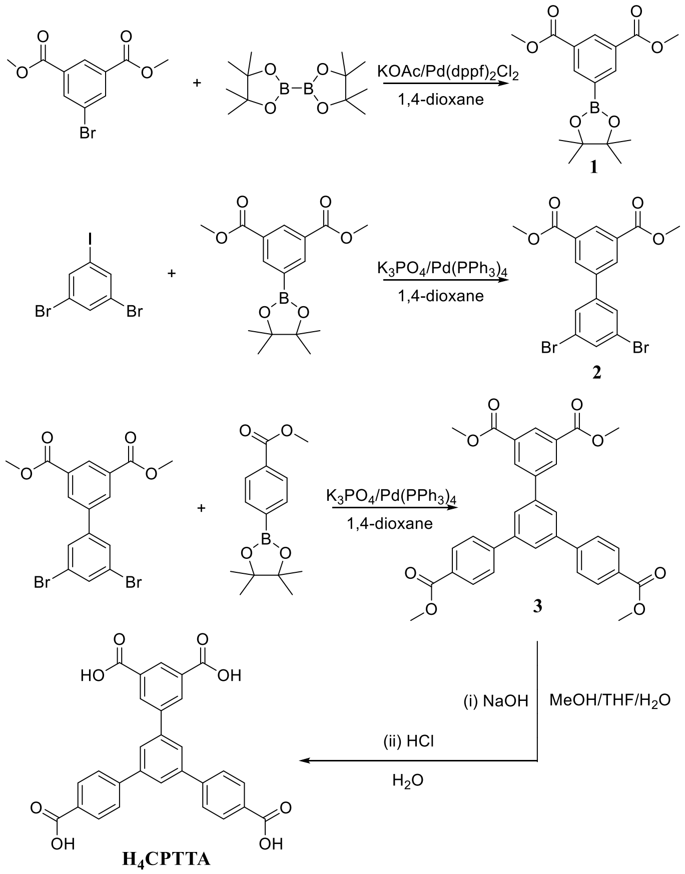

Supplementary Figure 22. Synthetic procedure for H4CPTTA.

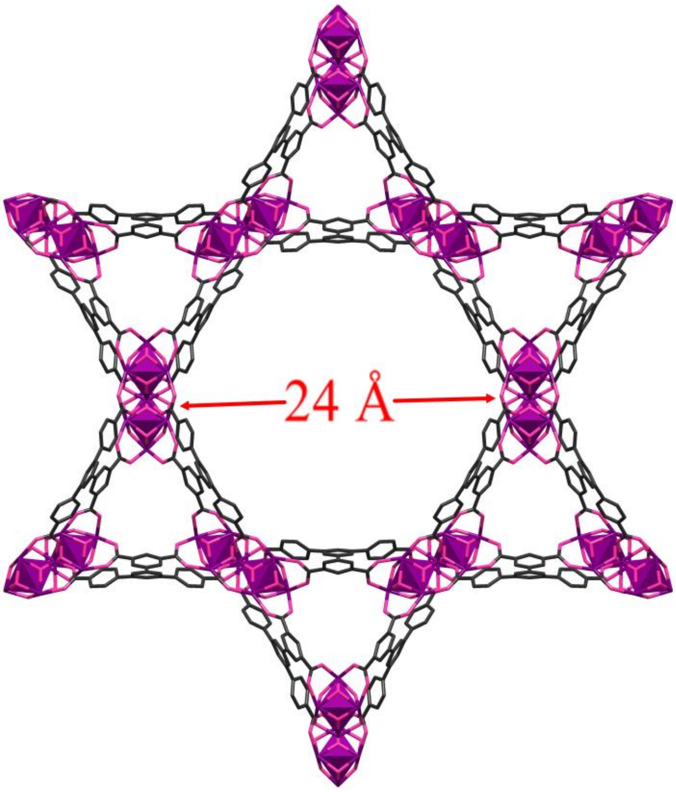

Supplementary Figure 23. Structure of BUT-17. One-dimensional channel in BUT-17 along *c* axis (color code: Zr, violet; C, black; and O, meagenta).

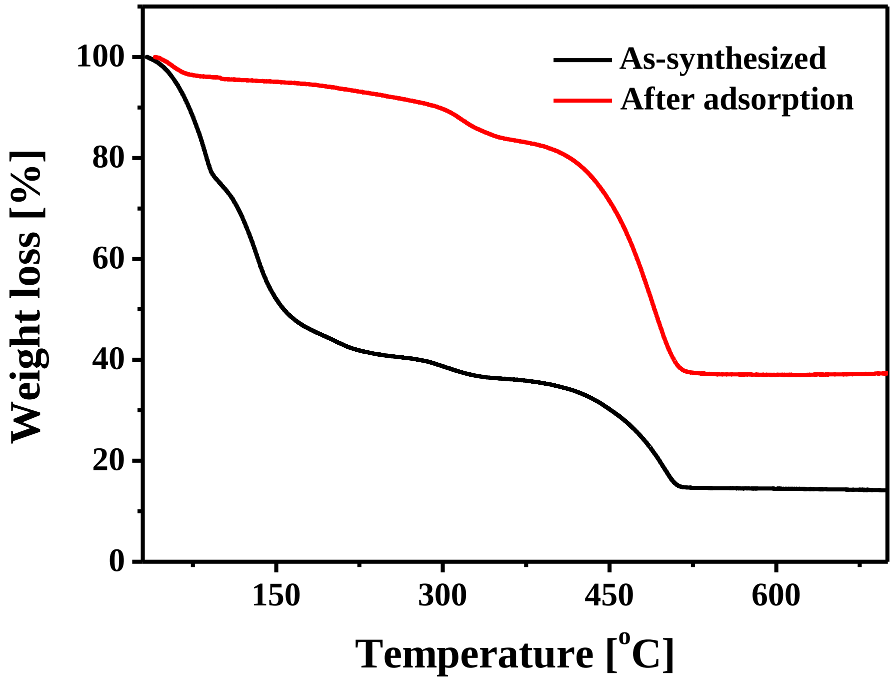

Supplementary Figure 24. TGA curves of BUT-17.

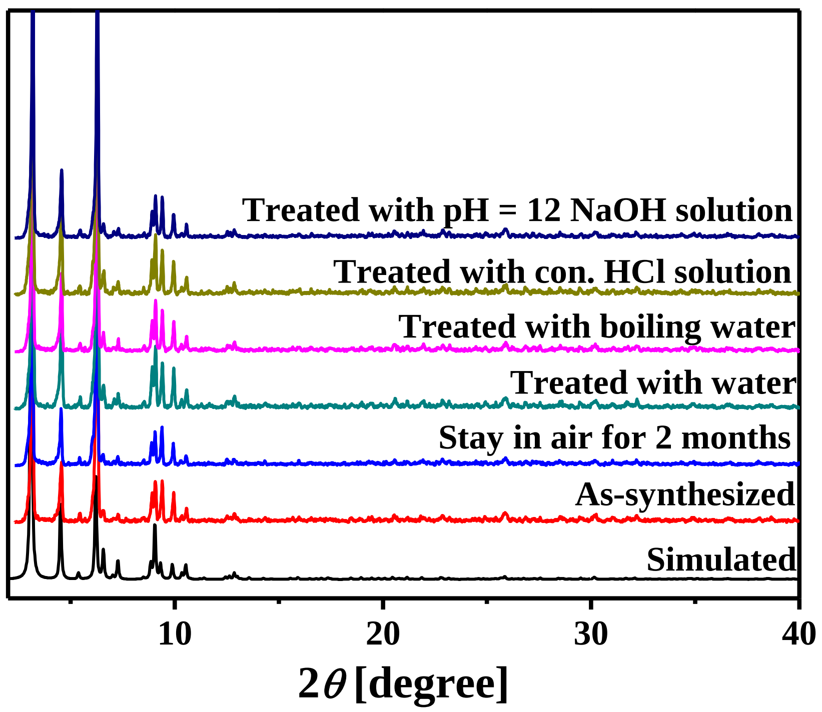

Supplementary Figure 25. PXRD patterns of BUT-17.

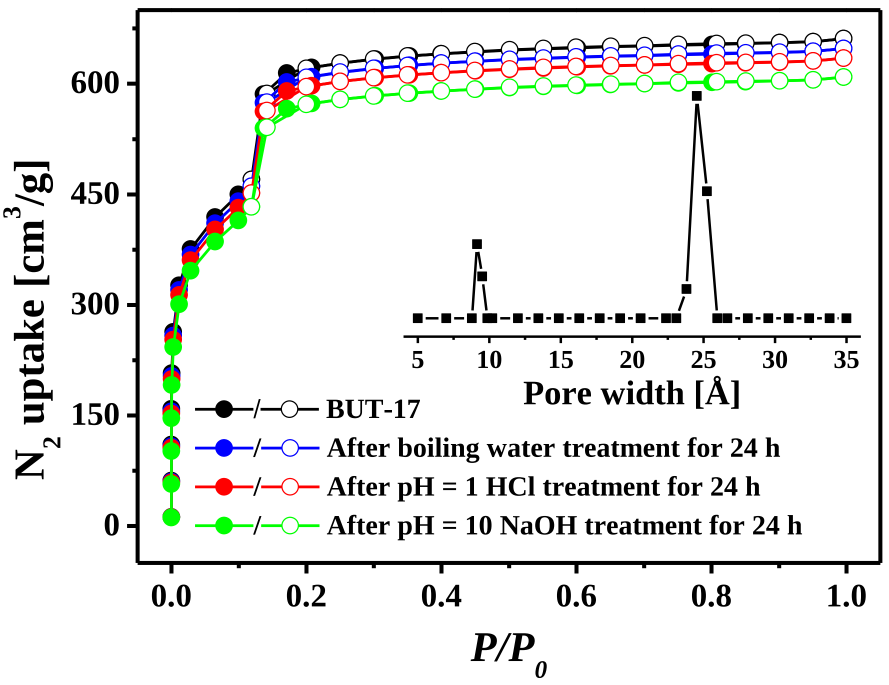

Supplementary Figure 26. N_2 adsorption/desorption isotherms of BUT-17 at 77 K (inset shows DFT pore size distribution evaluated by using the N_2 adsorption data).

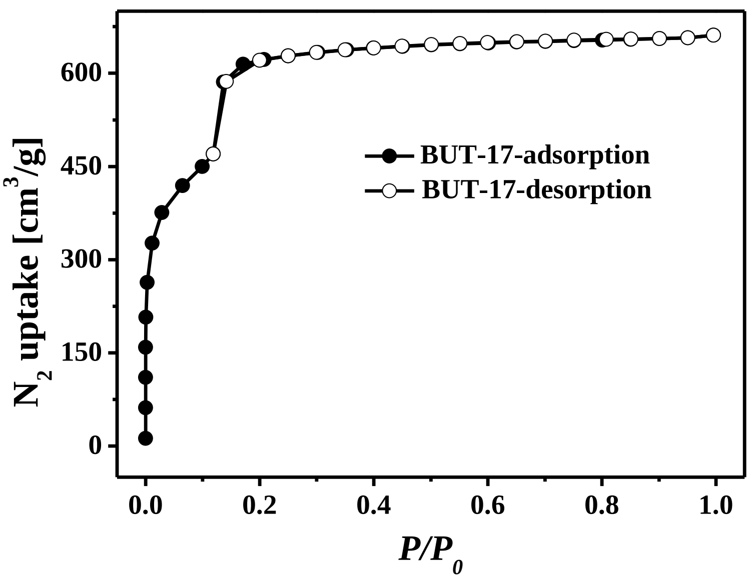

Supplementary Figure 27. Calculation of the BET surface area of BUT-17. N_2 adsorption/desorption isotherms of BUT-17 at 77 K.

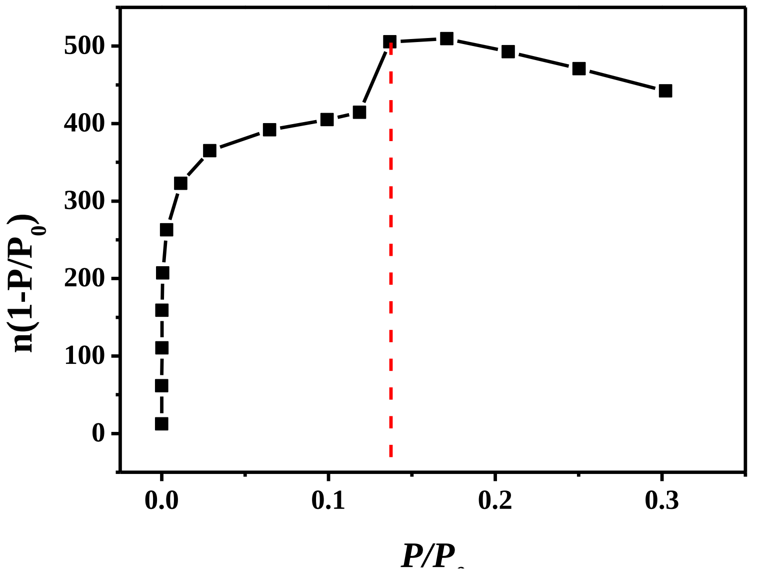

Supplementary Figure 28. Calculation of the BET surface area of BUT-17. Plot of n(1-P/P_0) vs. P/P_0 to determine the maximum P/P_0 used in the BET linear fit according to the first BET consistency criterion.

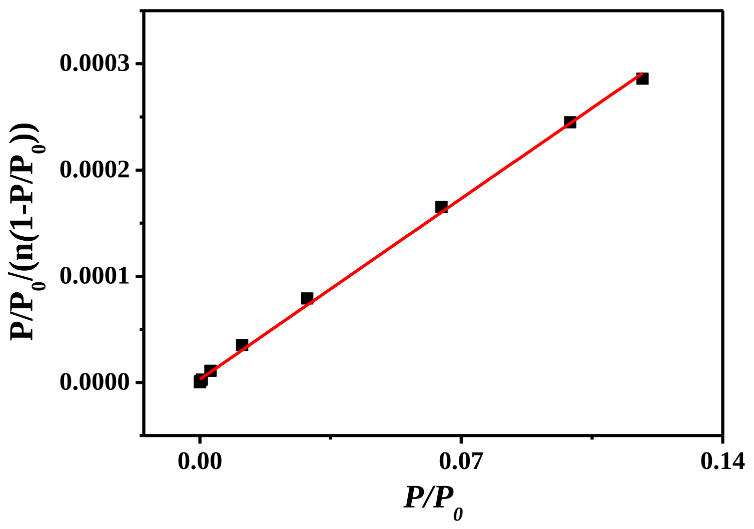

Supplementary Figure 29. Calculation of the BET surface area of BUT-17. Plot of $P/P_0/(n(1-P/P_0))$ vs. $P/P0$ to determine the BET surface area. The slope of the best fit line for $P/P_0 < 0.11$ is 0.00243, and the y-intercept is 2.92×10^{-6}, which satisfies the second BET consistency criterion. This results in a BET surface area of 1790 m^2 g^{-1}.

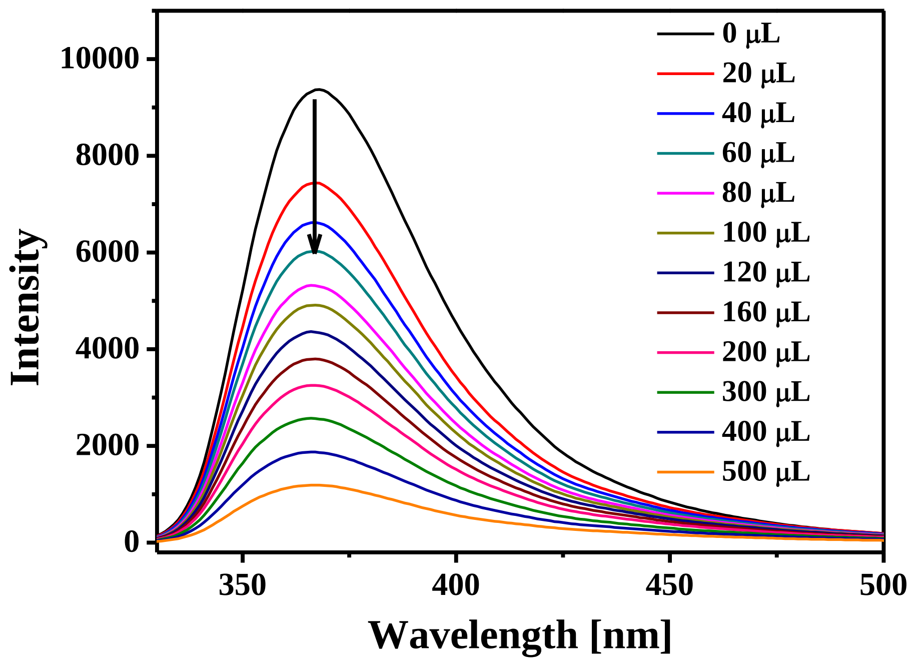

Supplementary Figure 30. Fluorescence-quenching titrations of BUT-17 by BCDD. Effect on the emission spectra of BUT-17 dispersed in hexane upon incremental addition of BCDD (100 ppm) hexane solution.

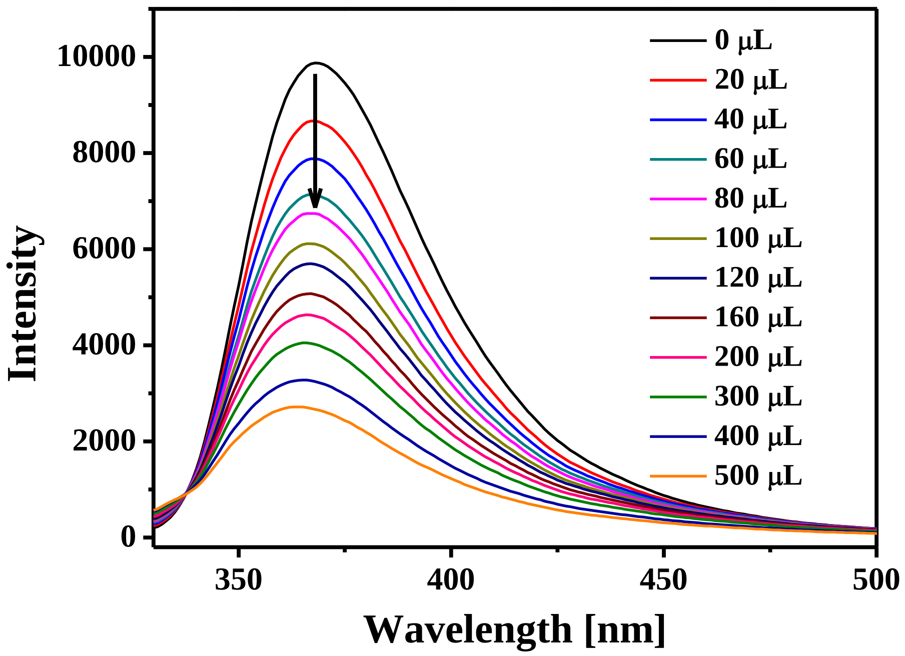

Supplementary Figure 31. Fluorescence-quenching titrations of BUT-17 by TCDD. Effect on the emission spectra of BUT-17 dispersed in hexane upon incremental addition of TCDD (100 ppm) hexane solution.

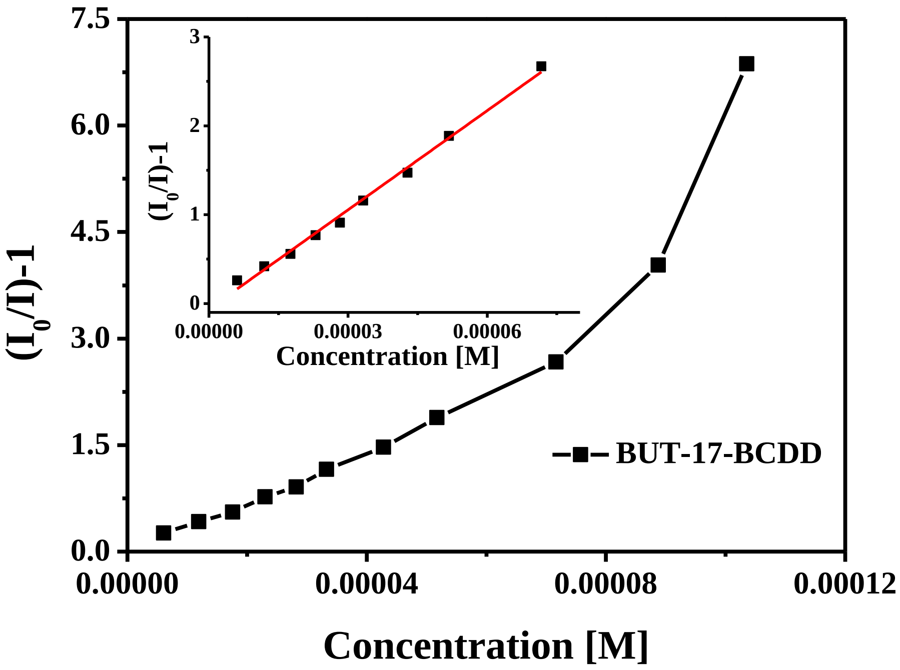

Supplementary Figure 32. SV plots of BCDD.

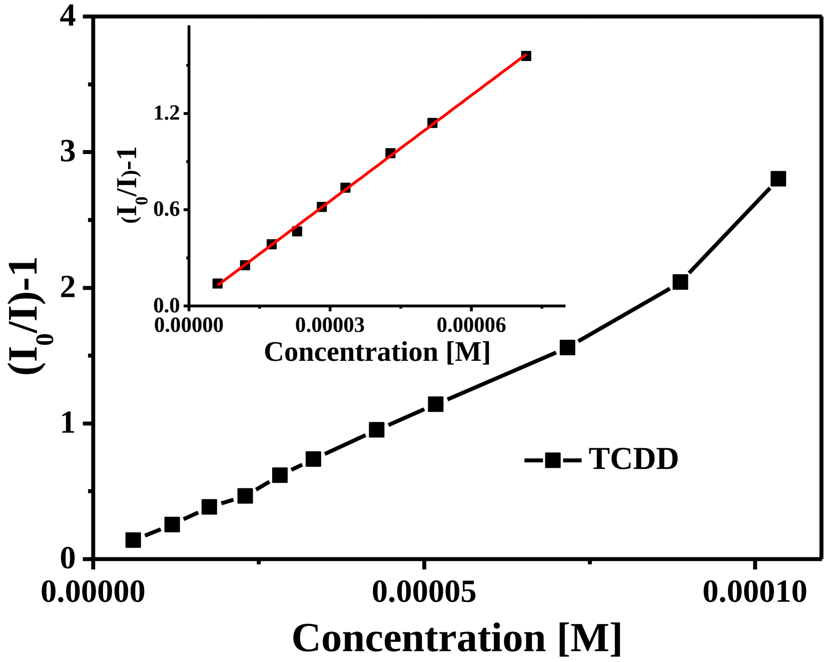

Supplementary Figure 33. SV plots of TCDD.

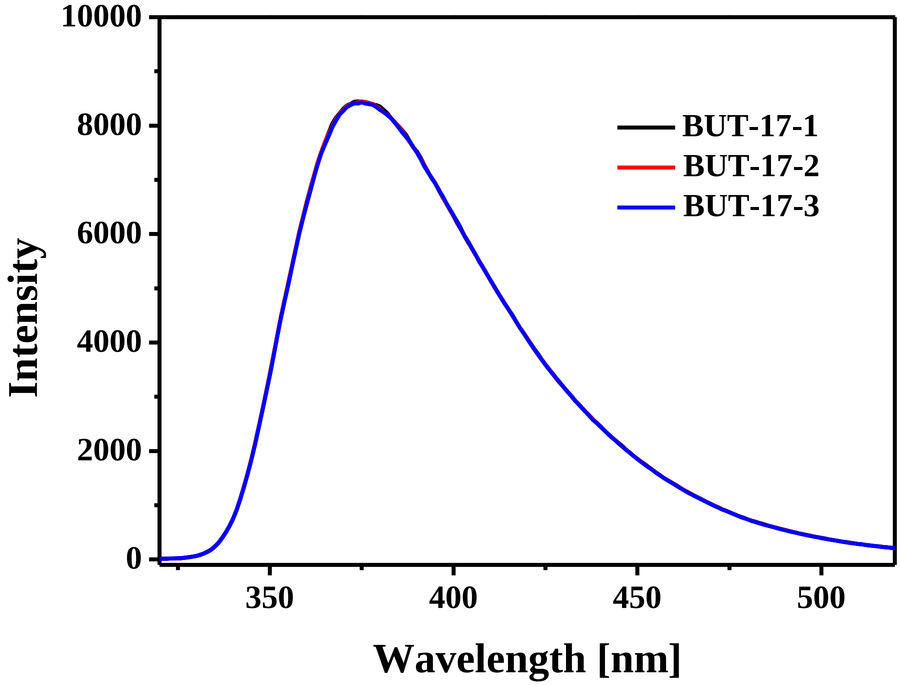

Supplementary Figure 34. Three repeated fluorescent measurements of blank solutions of BUT-17.

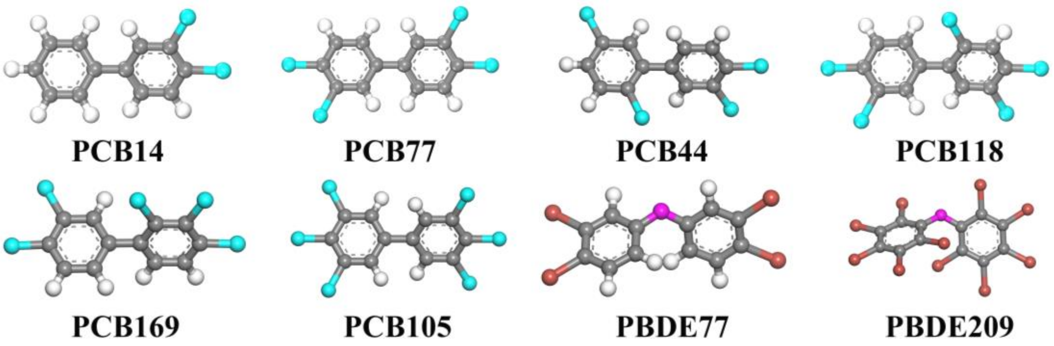

Supplementary Figure 35. Molecular structures of the selected interferents (color code: C, gray; O, magenta; Cl, turquoise, and Br, chocolate).

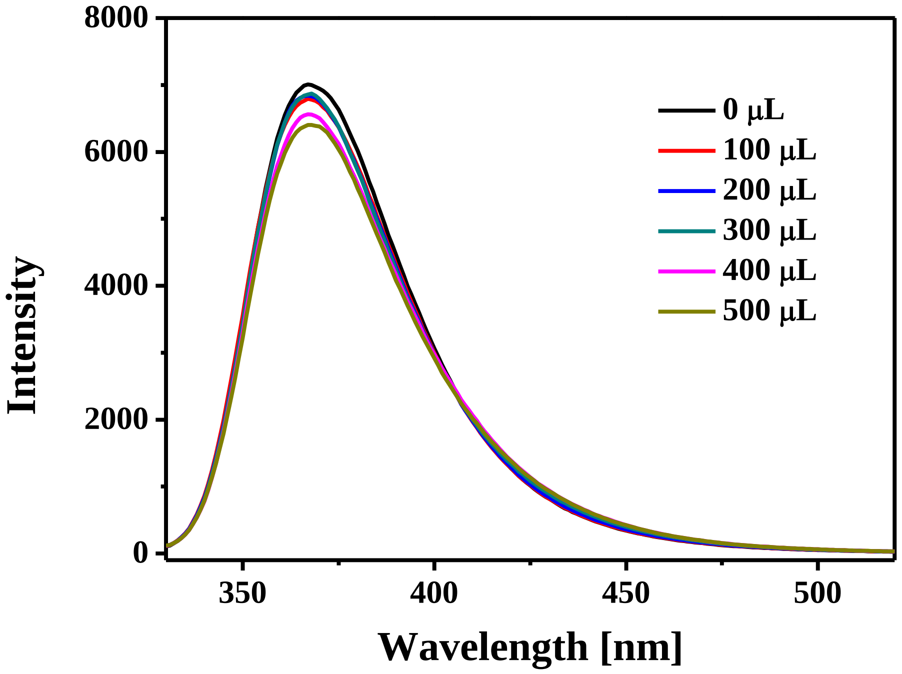

Supplementary Figure 36. Fluorescence-quenching titrations of BUT-17 by PCB14. Effect on the emission spectra of BUT-17 dispersed in hexane upon incremental addition of PCB14 (100 ppm) hexane solution.

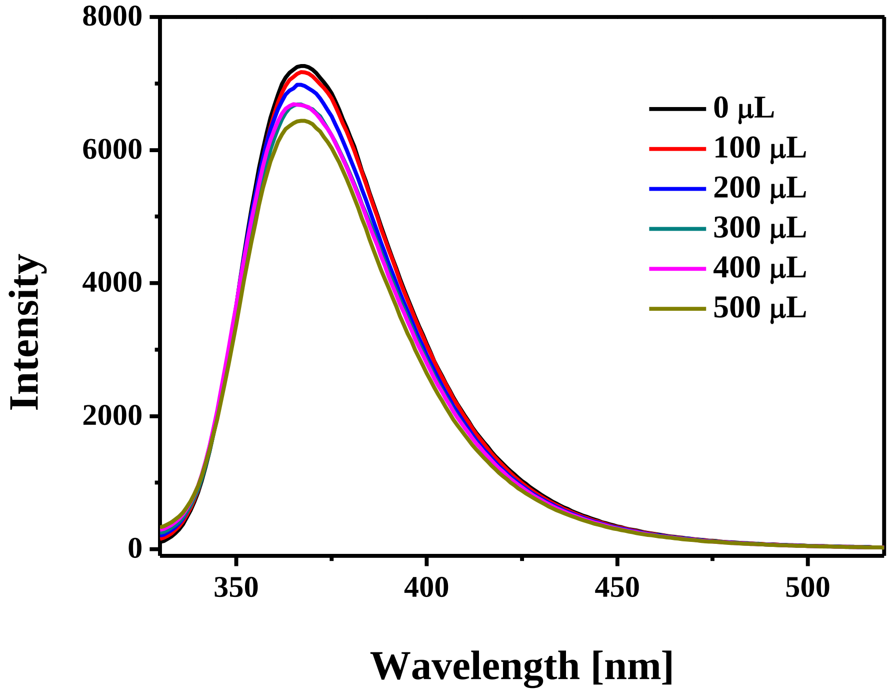

Supplementary Figure 37. Fluorescence-quenching titrations of BUT-17 by PCB77. Effect on the emission spectra of BUT-17 dispersed in hexane upon incremental addition of PCB77 (100 ppm) hexane solution.

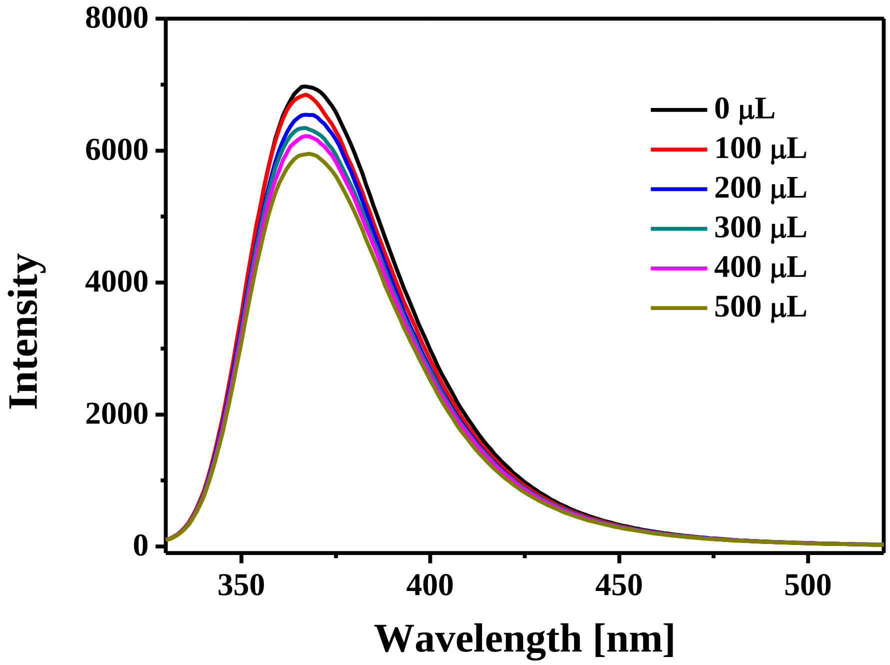

Supplementary Figure 38. Fluorescence-quenching titrations of BUT-17 by PCB44. Effect on the emission spectra of BUT-17 dispersed in hexane upon incremental addition of PCB44 (100 ppm) hexane solution.

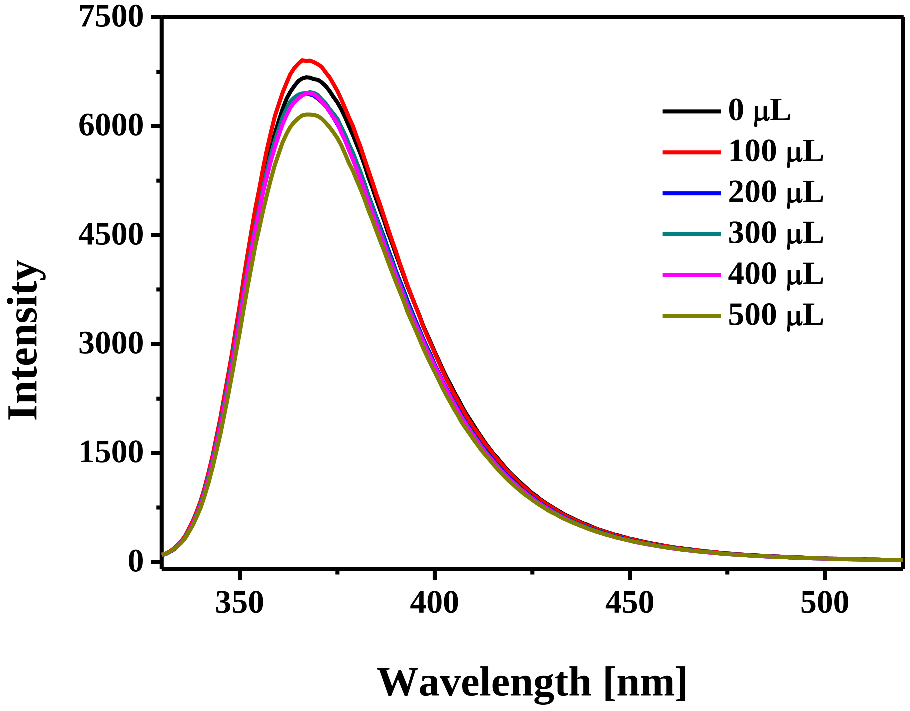

Supplementary Figure 39. Fluorescence-quenching titrations of BUT-17 by PCB118. Effect on the emission spectra of BUT-17 dispersed in hexane upon incremental addition of PCB118 (100 ppm) hexane solution.

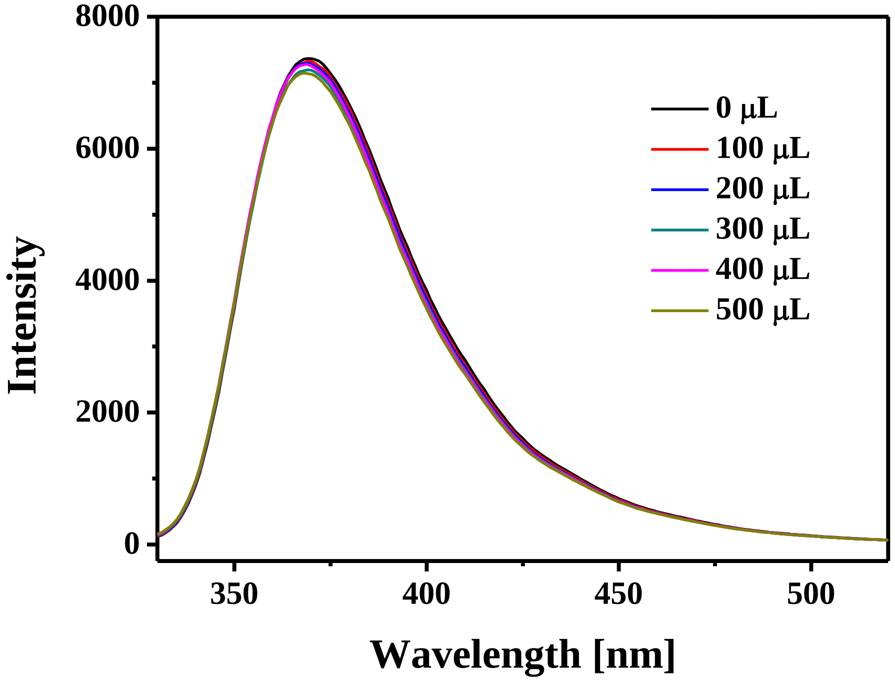

Supplementary Figure 40. Fluorescence-quenching titrations of BUT-17 by PCB105. Effect on the emission spectra of BUT-17 dispersed in hexane upon incremental addition of PCB105 (100 ppm) hexane solution.

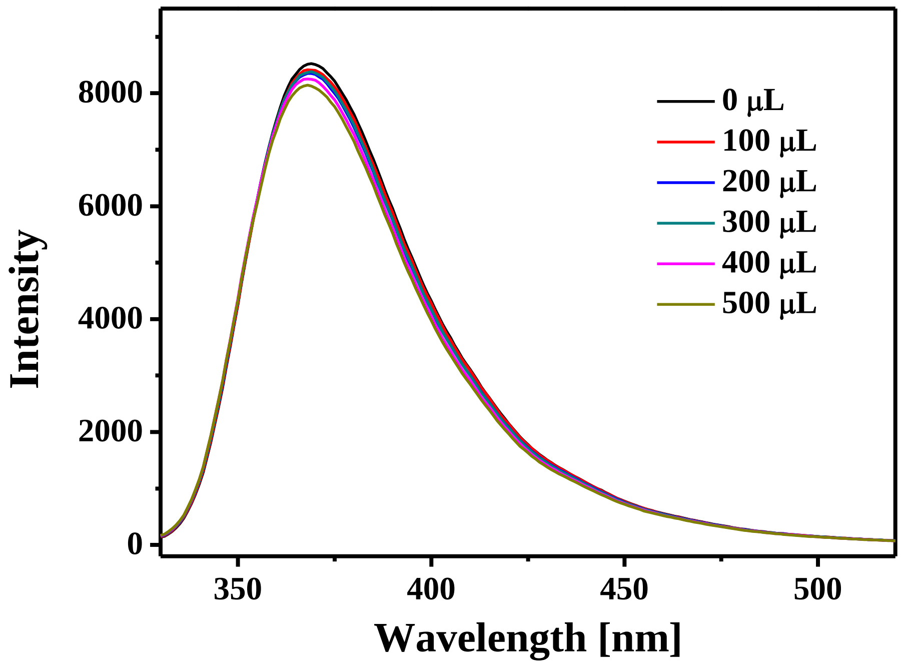

Supplementary Figure 41. Fluorescence-quenching titrations of BUT-17 by PCB169. Effect on the emission spectra of BUT-17 dispersed in hexane upon incremental addition of PCB169 (100 ppm) hexane solution.

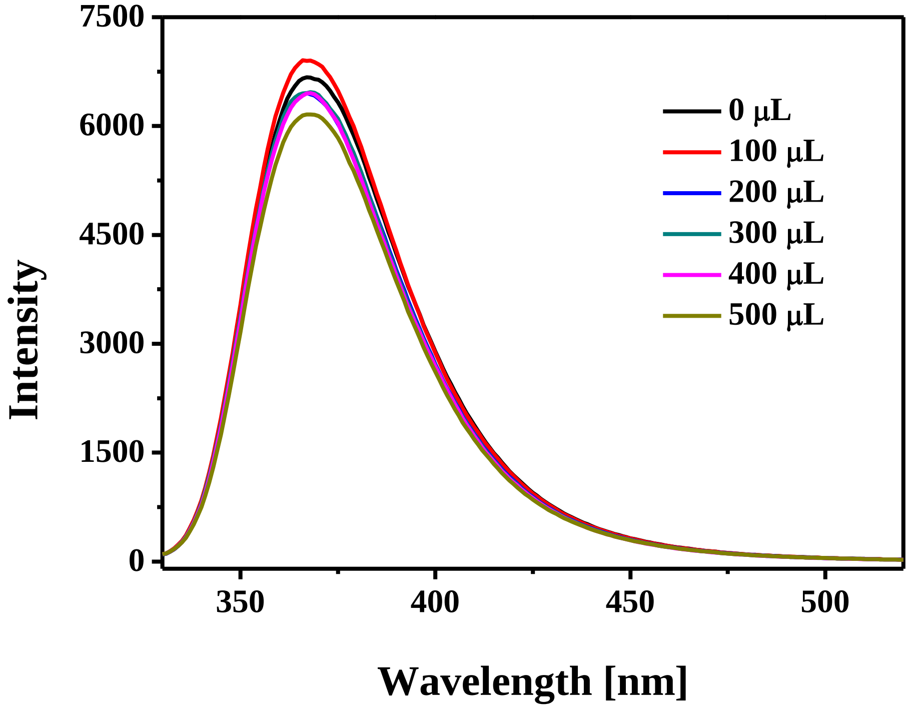

Supplementary Figure 42. Fluorescence-quenching titrations of BUT-17 by PBED77. Effect on the emission spectra of BUT-17 dispersed in hexane upon incremental addition of PBDE77 (100 ppm) hexane solution.

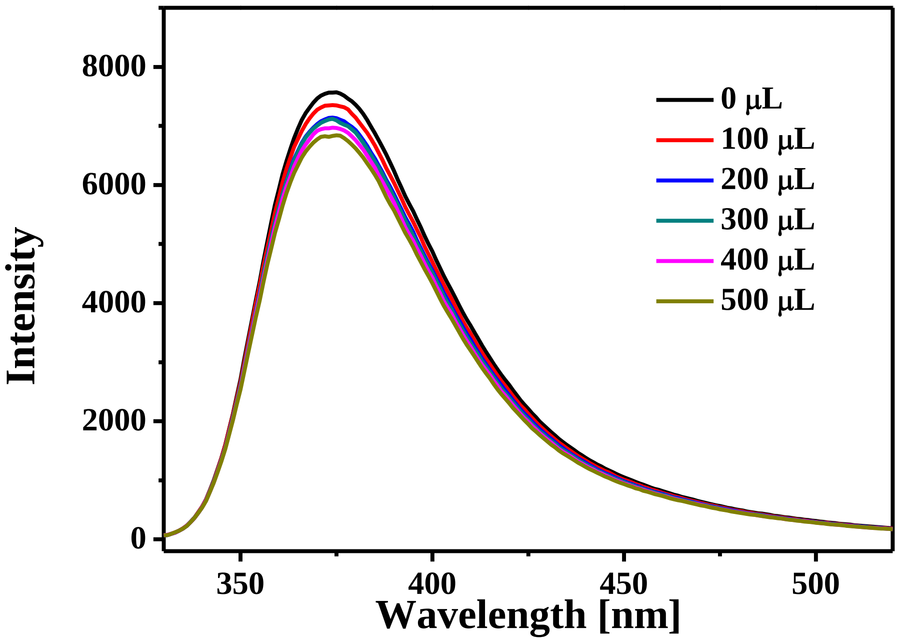

Supplementary Figure 43. Fluorescence-quenching titrations of BUT-17 by PBED209. Effect on the emission spectra of BUT-17 dispersed in hexane upon incremental addition of PBDE209 (100 ppm) hexane solution.

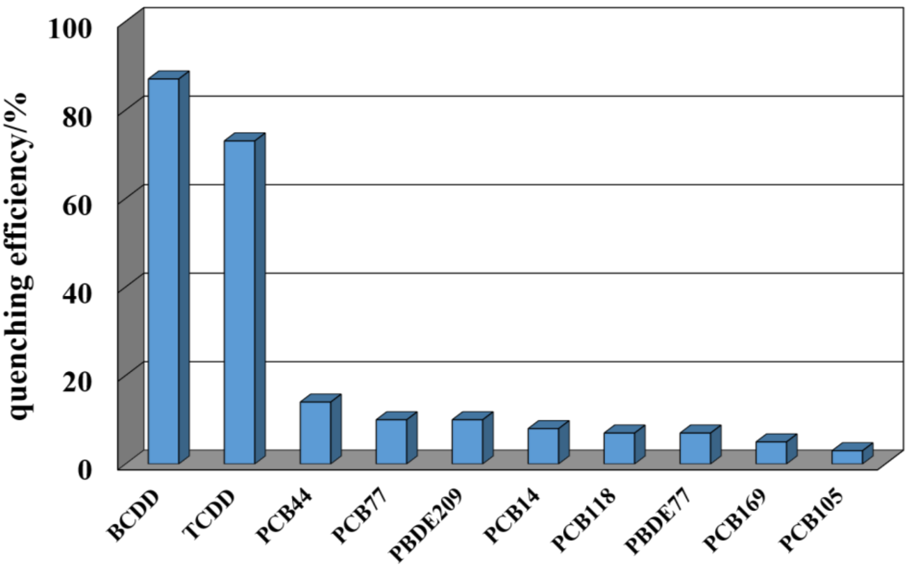

Supplementary Figure 44. Quenching efficiencies of BUT-17 by different analytes. Changes of the fluorescence of BUT-17 dispersed in hexane upon incremental addition of different analytes (100 ppm).

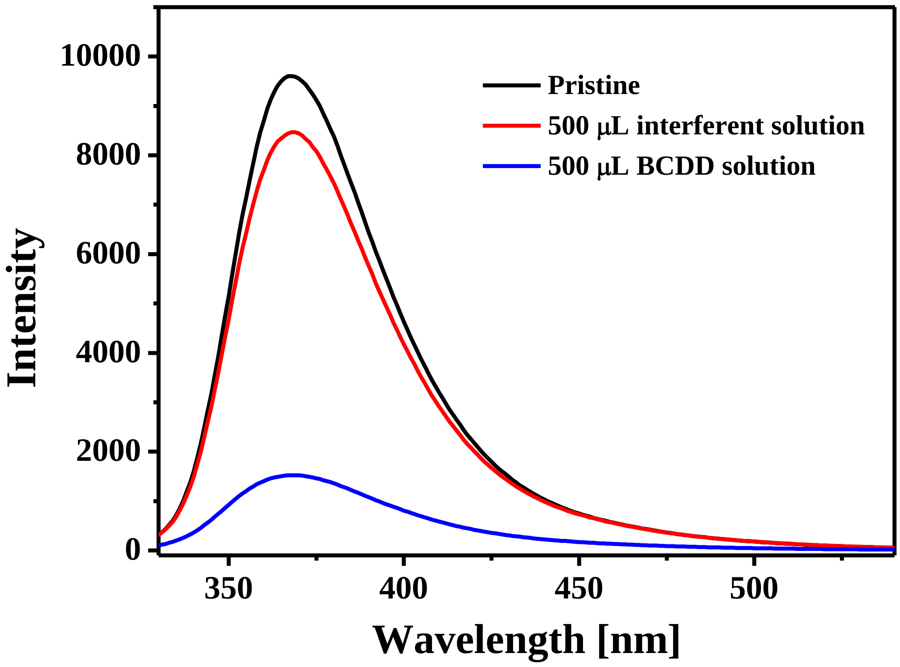

Supplementary Figure 45. Selective detection exploration. Fluorescent spectra of BUT-17 upon the addition of BCDD (500 μL, 100 ppm) in the presence of a background of 5 organic compound with similar structures (200 ppm for each interferent) in hexane, monitored at 320 nm.

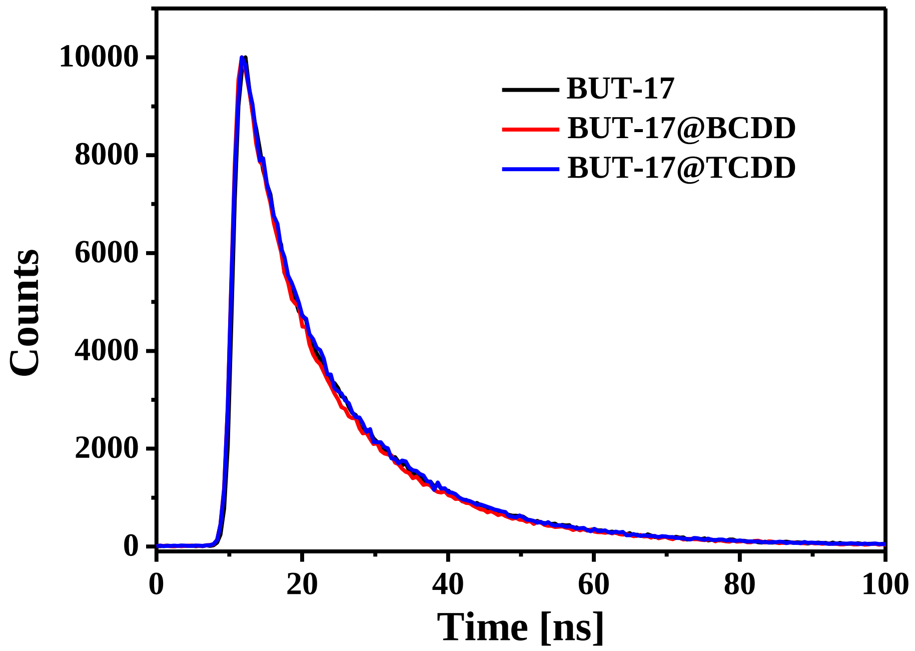

Supplementary Figure 46. Fluorescence lifetimes. Time-resolved fluorescence decay traces of BUT-17 and analyte loaded BUT-17 at room temperature.

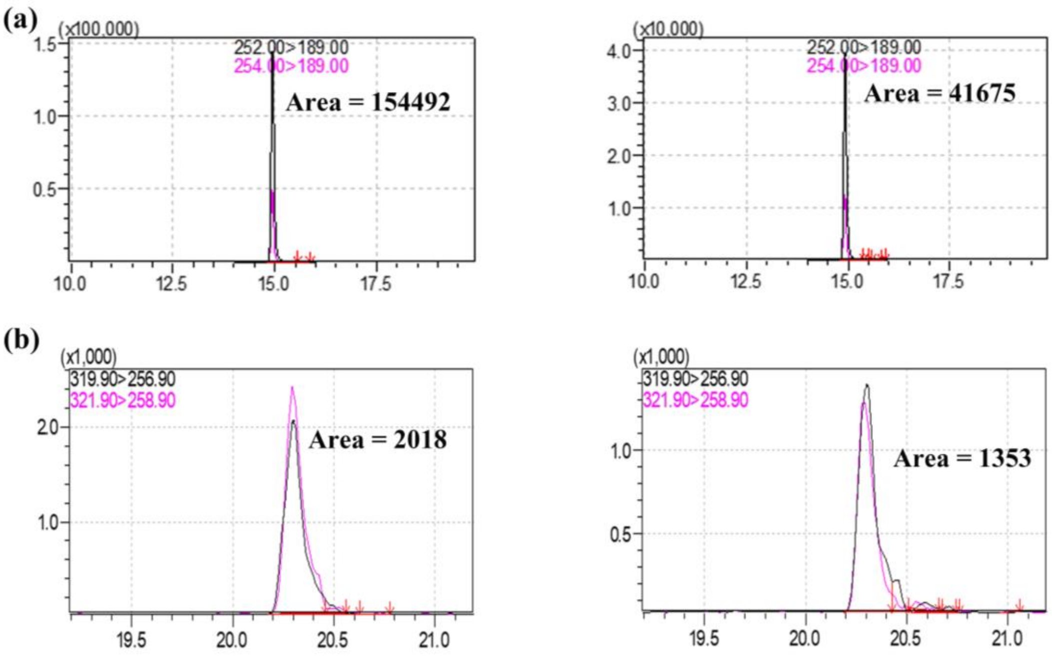

Supplementary Figure 47. Adsorption of BCDD and TCDD using BUT-17 as adsorbent. The areas of (a) BCDD before (left) and after (right) adsorption experiments, and (b) TCDD before (left) and after (right) adsorption experiments.

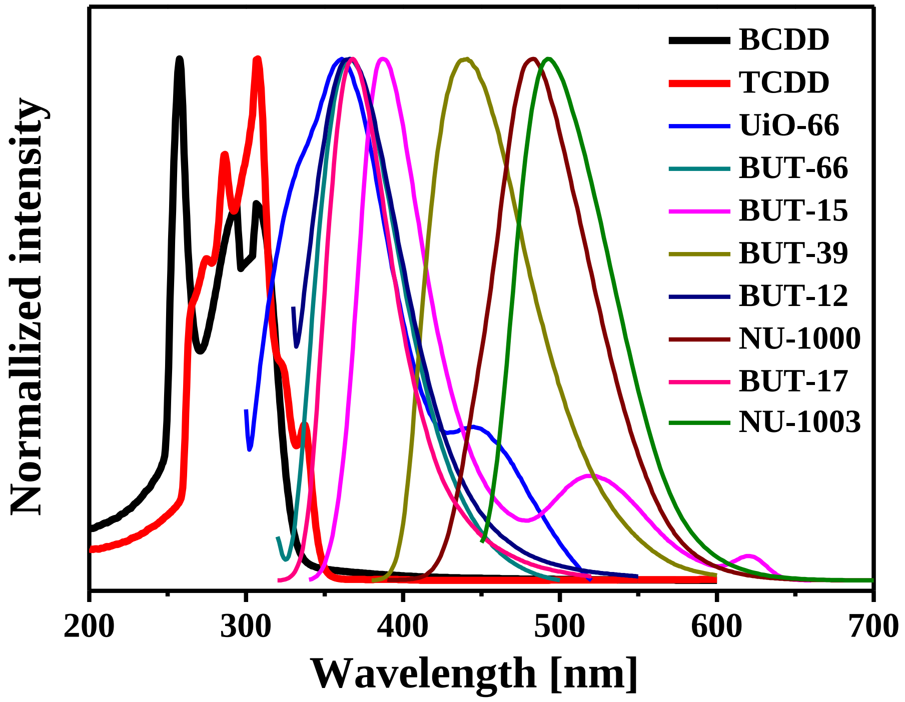

Supplementary Figure 48. The detection mechanism exploration. Spectral overlap between the absorption spectrum of BCDD, TCDD and the fluorescence emission spectra of BUT-17 and selected Zr-MOFs.

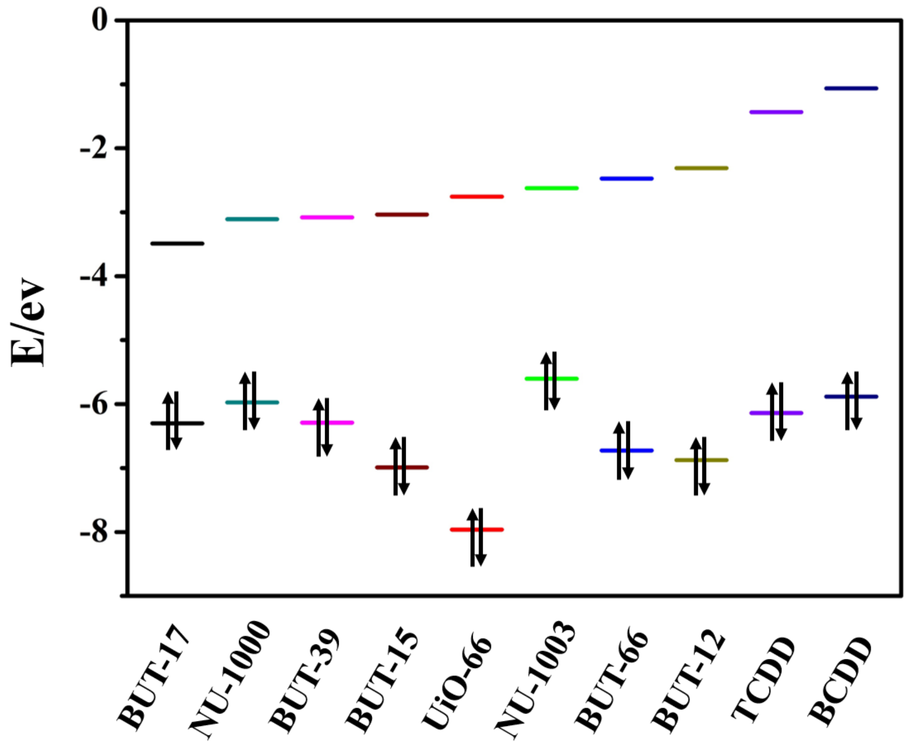

Supplementary Figure 49. The detection mechanism exploration. HOMO and LUMO energies for BCDD, TCDD, BUT-17, and selected Zr-MOFs arranged in increasing order of LUMO energies.

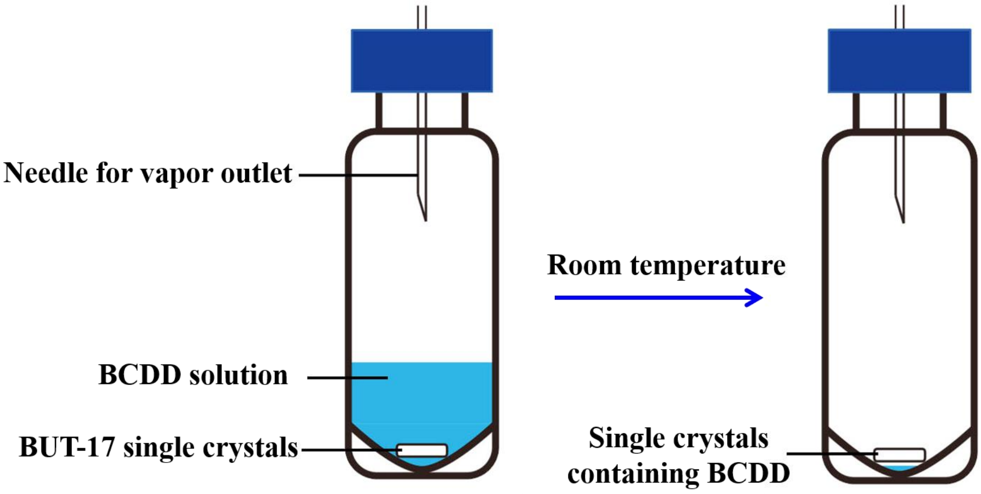

Supplementary Figure 50. Preparation of BUT-17@BCDD. Experimental settings for guest inclusion.

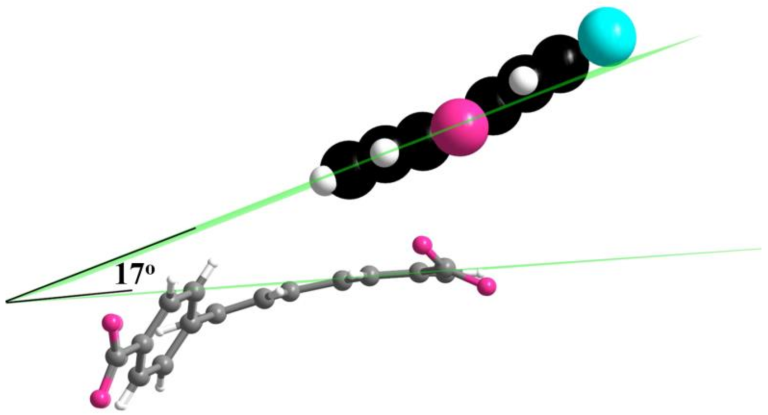

Supplementary Figure 51. Crystal structure of BUT-17@BCDD. The dihedral angle between BCDD molecule and the central benzene ring of $CPTTA^{4-}$ (Color code: C, gray; O, magenta; Cl, turquoise; and H, white).

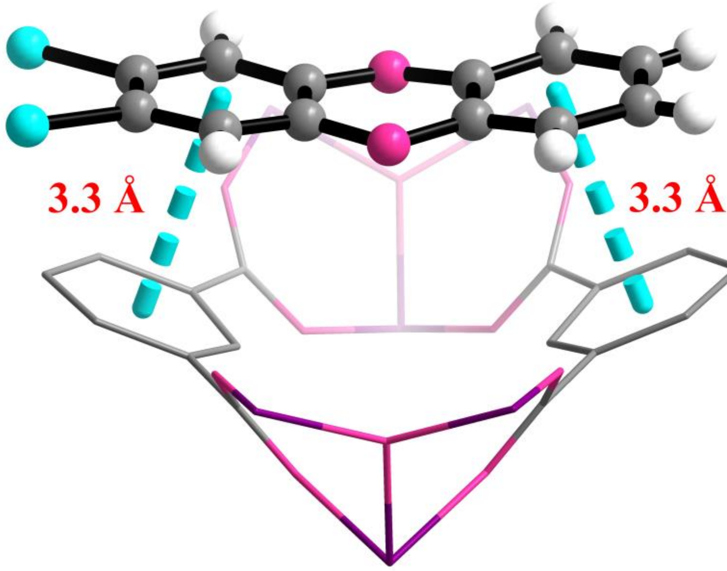

Supplementary Figure 52. Crystal structure of BUT-17@BCDD. Selected fragments highlighting the π-π stacking interactions in BCDD@BUT-17 (Color code: Zr, violet; C, gray; O, magenta; Cl, turquoise; and H, white).

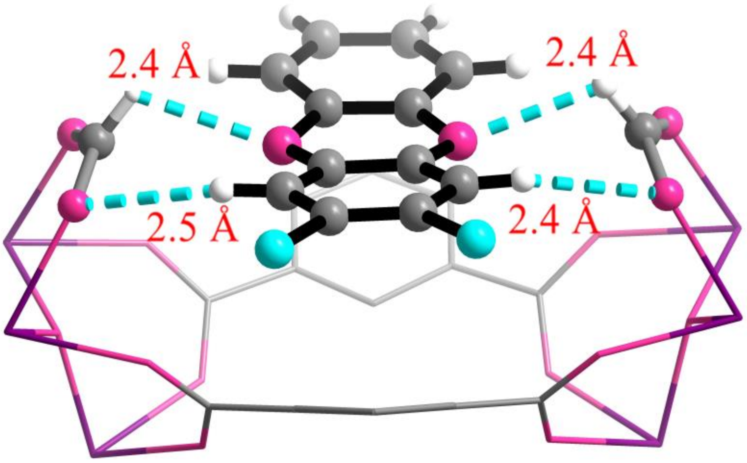

Supplementary Figure 53. Crystal structure of BUT-17@BCDD. Selected fragments highlighting the hydrogen bonding interactions in BCDD@BUT-17 (Color code: Zr, violet; C, gray; O, magenta; Cl, turquoise; and H, white).

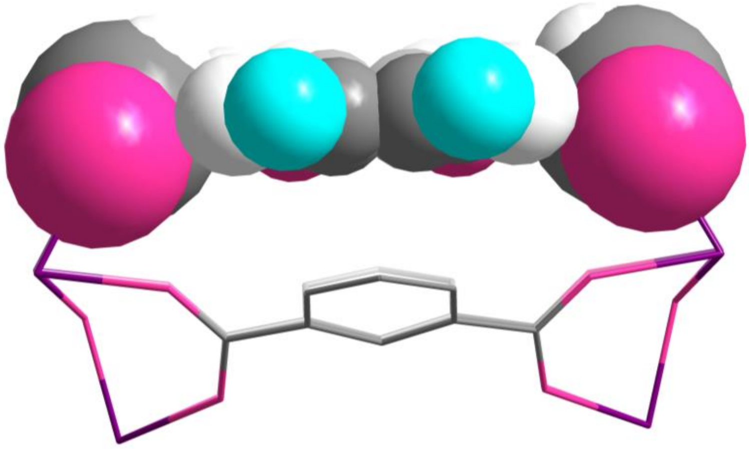

Supplementary Figure 54. Crystal structure of BUT-17@BCDD. Selected fragments highlighting the “molecule clip” in BCDD@BUT-17 (Color code: Zr, violet; C, gray; O, magenta; Cl, turquoise; and H, white).

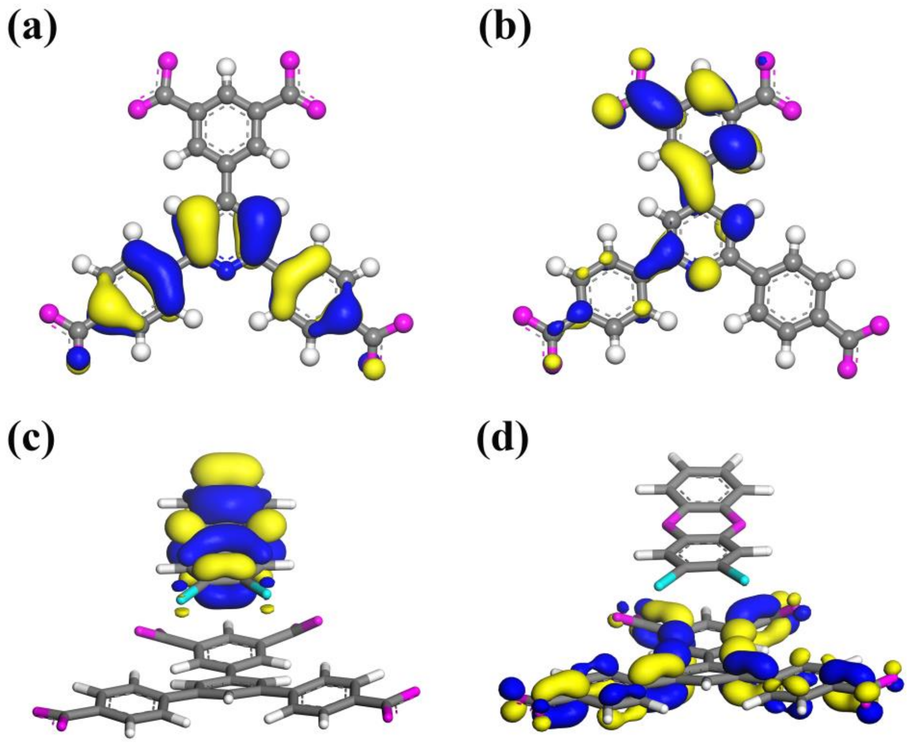

Supplementary Figure 55. DFT calculations. (a) The HOMO and (b) LUMO orbitals of BUT-17; (c) The HOMO and (d) LUMO orbitals of BUT-17@BCDD.

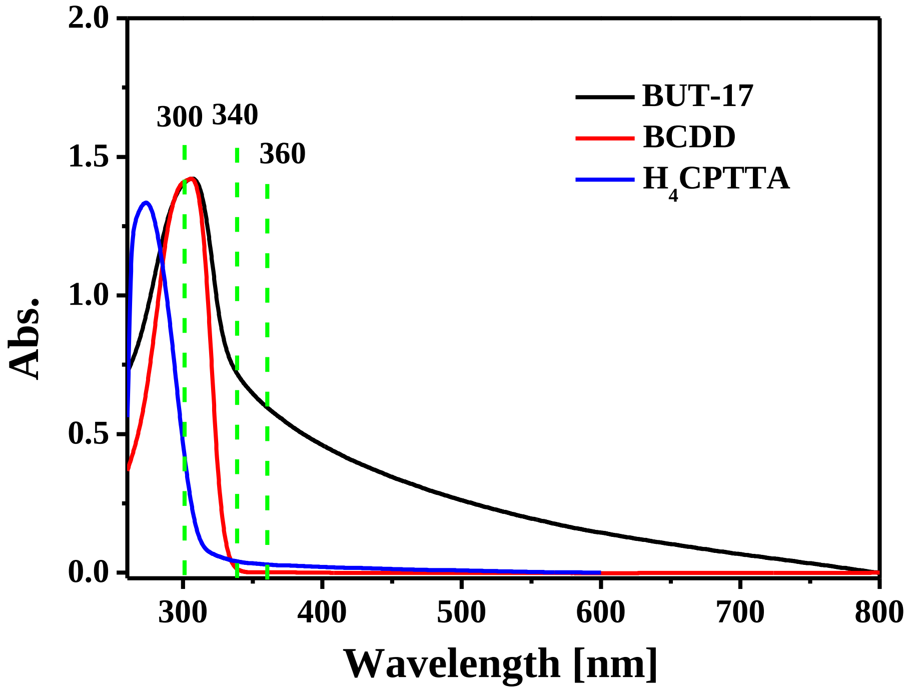

Supplementary Figure 56. Uv-vis spectra of BUT-17, BCDD, and H_4CPTTA in hexane.

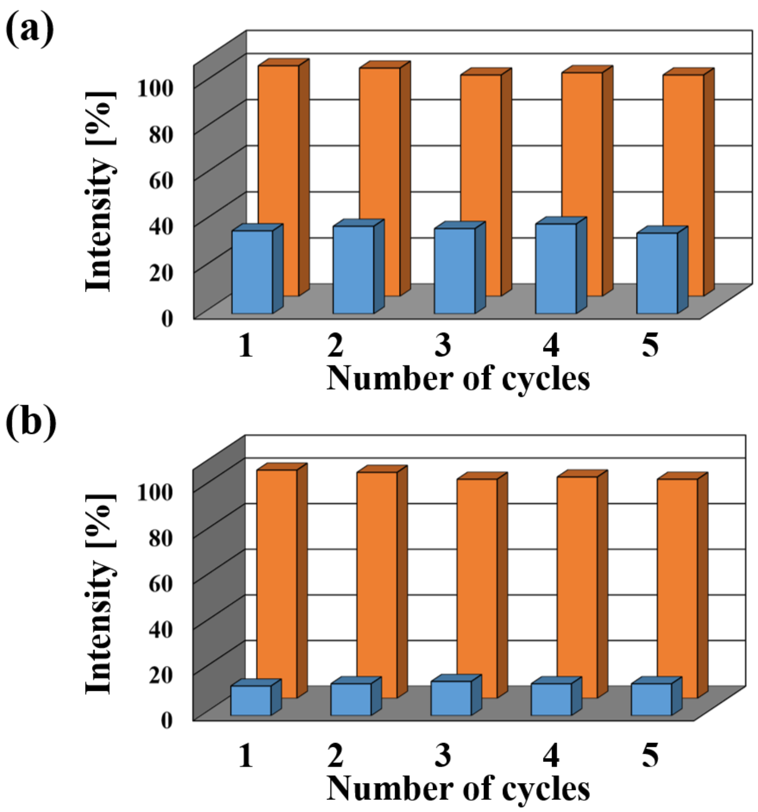

Supplementary Figure 57. Reproducibility of the quenching ability of **BUT-17** dispersed in hexane in the presence of 100 ppm (a) TCDD and (b) BCDD (The orange bars represent the initial fluorescence intensity and the blue bars represent the intensity upon addition a solution of 500 μL 100 ppm TCDD or BCDD in hexane).

**BUT-17** (CCDC: 1883541)	Before squeeze	After squeeze
Empirical formula	$C_{29}H_{15}O_{15.91}Zr_3$	$C_{29}H_{15}O_{15.91}Zr_3$
Formula weight	891.55	891.55
Measurement temperature	100.0(1) K	100.0(1) K
Crystal system	Hexagonal	Hexagonal
Space group	$P6_3/mmc$	$P6_3/mmc$
a (Å)	32.8745(3)	32.8745(3)
b (Å)	32.8745(3)	32.8745(3)
c (Å)	26.6031(3)	26.6031(3)
α (°)	90	90
β (°)	90	90
γ (°)	120	120
Volume(Å^3)	24898.9(5)	24898.9(5)
Z	12	12
Calculated density(g/cm^3)	0.735	0.714
Absorption coefficient (mm^{-1})	3.319	3.294
Independent reflections ($I > 2\sigma(I)$)	8020 [R(int) = 0.0786]	8020 [R(int) = 0.0717]
F(000)	5396	5235
Reflections collected	88495	88495
Completeness to theta = 133.12°	100%	100%
θ range for data collection	6.644-134.14	6.644-134.14
Data/restraints/parameters	8020/0/234	8020/0/234
Limiting indices	$-38 \le h \le 39$<br>$-28 \le k \le 38$<br>$-31 \le l \le 23$	$-38 \le h \le 39$<br>$-28 \le k \le 38$<br>$-31 \le l \le 23$
Goodness-of-fit on F^2	1.570	1.066
R_1 ^{a},wR_2 ^{b} [$I > 2\sigma(I)$]	R_1 = 0.1046, wR_2 = 0.3315	R_1 = 0.0453, wR_2 = 0.1316
R_1 ^{a}, wR_2 ^{b} (all data)	R_1 = 0.1293, wR_2 = 0.3681	R_1 = 0.0564, wR_2 = 0.1381
Largest diff. peak and hole (e/Å^3)	6.25 and –1.18	1.36 and –0.75

^{a} $R_1 = \Sigma(||F_0|-|F_C||)/\Sigma|F_0|$.

^{b} $wR_2 = [\Sigma w(|F_0|^2-|F_C|^2)^2/\Sigma w(F_0^2)]^{1/2}$.

Supplementary Table 1. Crystal data and structure refinement for **BUT-17**.

**BUT-17** (CCDC: 1883542)	Before squeeze	After squeeze
Empirical formula	$C_{59}H_{29.67}Cl_{0.67}O_{36.07}Zr_6$	$C_{59}H_{29.67}Cl_{0.67}O_{36.07}Zr_6$
Formula weight	1886.51	1886.51
Measurement temperature	100.00(10)	100.00(10)
Crystal system	Hexagonal	Hexagonal
Space group	$P6_3/mmc$	$P6_3/mmc$
a (Å)	32.8142(5)	32.8142(5)
b (Å)	32.8142(5)	32.8142(5)
c (Å)	26.9986(10)	26.9986(10)
α (°)	90	90
β (°)	90	90
γ (°)	120	120
Volume(Å^3)	25176.5(12)	25176.5(12)
Z	6	6
Calculated density(g/cm^3)	0.747	0.747
Absorption coefficient (mm^{-1})	3.390	3.390
Independent reflections ($I > 2\sigma(I)$)	7607 [R(int) = 0.1143]	7607 [R(int) = 0.1043]
F(000)	5541	5541
Reflections collected	115150	115150
Completeness to theta = 130.324°	100%	100%
θ range for data collection	8.482-130.324°	8.482-130.324°
Data/restraints/parameters	7607/354/270	7607/354/270
Limiting indices	$-38 \le h \le 38$<br>$-38 \le k \le 38$<br>$-31 \le l \le 21$	$-38 \le h \le 38$<br>$-38 \le k \le 38$<br>$-31 \le l \le 21$
Goodness-of-fit on F^2	1.075	1.039
R_1 ^{a},wR_2 ^{b} [$I > 2\sigma(I)$]	R_1 = 0.0686, wR_2 = 0.1662	R_1 = 0.0562, wR_2 = 0.1514
R_1 ^{a}, wR_2 ^{b} (all data)	R_1 = 0.1023, wR_2 = 0.2001	R_1 = 0.0710, wR_2 = 0.1601
Largest diff. peak and hole (e/Å^3)	1.28 and –1.07	1.66 and –1.13

^{a} $R_1 = \Sigma(||F_0|-|F_C||)/\Sigma|F_0|$.

^{b} $wR_2 = [\Sigma w(|F_0|^2-|F_C|^2)^2/\Sigma w(F_0^2)]^{1/2}$.

Supplementary Table 2. Crystal data and structure refinement for **BUT-17@BCDD**.

MOF	τ1 (ns)	τ2 (ns)	B1 (%)	B2 (%)	τ(ns)
BUT-17	7.472	18.15	41.56	58.44	13.7
BUT-17@BCDD	5.961	17.05	30.60	69.40	13.6
BUT-17@TCDD	6.991	17.41	38.02	61.98	13.4

Supplementary Table 3. Fluorescence decay parameters of BUT-17 and analyte loaded BUT-17 at room temperature

**Analytes**	**HUMO (ev)**	**LUMO (ev)**	**Band Gap (ev)**
BUT-17	-6.301	-3.490	2.811
NU-1000	-5.974	-3.110	2.864
BUT-39	-6.290	-3.080	3.210
BUT-15	-6.988	-3.035	3.953
UiO-66	-7.961	-2.757	5.204
BUT-66	-6.724	-2.474	4.250
BUT-12	-6.876	-2.311	4.565
NU-1003	-5.602	-2.624	2.978
TCDD	-6.140	-1.435	4.705
BCDD	-5.883	-1.065	4.818

Supplementary Table 4. HOMO and LUMO energies calculated for selected MOFs, BUT-17, TCDD, and BCDD at B3LYP/6-31G* level.

**Analytes**	**Wavelength (nm)**
BUT-17	300
NU-1000	370
BUT-39	280
BUT-15	310
UiO-66	280
BUT-66	366
BUT-12	310
NU-1003	430

Supplementary Table 5. The excitation wavelengths of BUT-17 and selected Zr-MOFs.
